# Camel Milk Exosomes Alleviate Doxorubicin-Induced Cardiotoxicity by Regulating Apoptosis and Autophagy via the NF-κB and MAPK Pathways

**DOI:** 10.3390/biology15181604

**Published:** 2026-09-11

**Authors:** Zhihua Wang, Qi Tian, Fanhua Meng, Shenyuan Wang, Lu Li, Junwei Cao

**Affiliations:** 1 College of Life Sciences, Inner Mongolia Agricultural University, Hohhot 010011, China; 2 Inner Mongolia Key Laboratory of Biomanufacturing Technology, Hohhot 010011, China; 3 Inner Mongolia Endemic Livestock Biotechnology Innovation Team, Hohhot 010011, China

**Keywords:** camel milk exosomes, doxorubicin, NF-κB, MAPK, autophagy, apoptosis

## Abstract

Doxorubicin (Dox)-induced cardiotoxicity (DIC) is a major clinical challenge in cancer therapy. Camel milk exosomes (CMEs) have been applied in anti-tumor treatments as they have a variety of effects, including on inflammation, oxidative stress, metastasis, and apoptosis. However, their role in DIC treatment remains incompletely understood. This research was designed to evaluate the protection provided by CMEs against DIC. The DIC mice were treated with Dox intraperitoneally and divided into a model group and groups treated with different doses of CMEs. Dox-induced H9c2 cell injury was also established and divided into a model group and groups treated with different concentrations of CMEs. The evaluation parameters in vitro included H9c2 cell viability, reactive oxygen species (ROS), mitochondria, and apoptotic cells. The apoptosis and autophagy markers, as well as the nuclear factor kappa B (NF-κB) and mitogen-activated protein kinase (MAPK) pathways, were assessed via Western blotting both in vivo and in vitro. In addition, transcriptome sequencing of cardiac tissue was also applied to investigate the related mechanisms. Our results indicate that CMEs significantly attenuated the cell viability reduction, apoptosis, and ROS production in H9c2 cells caused by Dox. CMEs also regulated autophagy, inhibited apoptosis, and inhibited the NF-κB and MAPK pathways. In conclusion, our findings demonstrate that CMEs exert a cardiac protective effect against DIC by inhibiting apoptosis and regulating autophagy via NF-κB and MAPK signaling pathways.

## Simple Summary

Doxorubicin (Dox)-induced cardiotoxicity (DIC) is a major obstacle in cancer treatment. Although camel milk exosomes (CMEs) have several pharmacological effects, their function in DIC remains unclear. By establishing Dox-induced C57BL/6 mice and H9c2 cell injury models, we found that CMEs exert a cardiac protective effect by alleviating oxidative stress and cardiomyocyte apoptosis and regulating autophagy via inhibition of the NF-κB and MAPK signaling pathways.

## 1. Introduction

Doxorubicin (Dox)-induced cardiotoxicity (DIC) is a major clinical limitation of Dox, which is a potent chemotherapeutic agent used in cancer treatment, including leukemia, lymphoma, and solid tumors [1]. DIC in clinical practice is mainly characterized by left ventricular dysfunction, arrhythmias, and eventually congestive heart failure [2]. Several mechanisms are associated with DIC, including oxidative stress, calcium dysregulation, mitochondrial damage, inflammation, and programmed cell death pathways [3]. With respect to these mechanisms, apoptosis has been thought to play a central role in DIC development, as well as in autophagy [4]. It has been reported that during DIC development, the intrinsic apoptosis pathway is activated via the up-regulation of pro-apoptotic factors, the down-regulation of anti-apoptotic factors, and the release of cytochrome C, which ultimately causes the occurrence of caspase-9 and caspase-3 cascades [5]. The autophagy occurrence playing a protective effect begins with the activation of unc-51-like autophagy-activating kinase 1 (ULK1) via AMP-activated protein kinase (AMPK), whereas persistent injury may lead to mammalian target of rapamycin (mTOR)-independent excessive autophagy and cause cell death [6]. During DIC development, persistent oxidative stress initiates massive cardiomyocyte apoptosis and a robust inflammatory reaction [7]. Moreover, inflammatory signaling pathways, particularly the tumor necrosis factor-α (TNF-α) and mitogen-activated protein kinase (MAPK) pathways (including p38, JNK, and ERK), participate in the progression of DIC by promoting cytokine release and oxidative enhancement [8]. Additionally, several other signaling pathways, such as the phosphatidylinositol 3-kinase (PI3K), Janus kinase (JAK), and signal transducer and activator of transcription (STAT) pathways, also participate in DIC occurrence [9]. As of now, dexrazoxane is still the only FDA-approved cardiac protective agent for DIC; however, its application is limited by a number of side effects, including myelosuppression and secondary malignancies [10]. Developing novel treatments to mitigate DIC is therefore of great importance.

Extracellular vesicles (EVs) are secreted by nearly all types of cells in the size range of 40~150 nm, which are transported in the form of microvesicles and exosomes [11,12]. In recent years, several studies have focused on natural products with bio-activity in DIC treatment, including exosomes [13]. As natural extracellular nanovesicles, exosomes range in size from 30 nm to 150 nm in diameter, and have emerged as promising therapeutic agents for cardiovascular diseases, including DIC [14]. Several studies have demonstrated that exosomes can be obtained from various sources, including mesenchymal stem cells, cardiac progenitor cells, and milk [15]. In addition, exosomes have been reported to play a protective role against DIC via the inhibition of apoptosis, inflammatory and oxidative reactions, etc. [13]. Camel milk (CM) is gaining more and more attention as a functional food with well-known antioxidant, immunoregulation, and anti-inflammatory effects [16]. Camel milk exosomes (CME) obtained from CM have unique stability, high bioavailability, and low immunogenicity [17]. CMEs are nano-sized extracellular vesicles, which are full of proteins, lipids, and microRNAs [17]. It has been reported that CMEs have effects on colorectal cancer [18], hyperglycemia [19], colitis [20], diabetic nephropathy [21], etc. We hypothesize that CMEs exert cardiac-protective effects by modulating the autophagy cascade. In addition, CMEs are predicted to suppress over-autophagy triggered by Dox and to preserve the protective autophagy associated with the AMPK/ULK1 pathway to maintain cardiomyocyte survival. However, the protective effect of CMEs against cardiac diseases such as DIC and its underlying mechanisms remain unexplored. In this study, we aimed to investigate the protective effects of CMEs against DIC and to elucidate the potential mechanisms involved.

## 2. Materials and Methods

### 2.1. Preparation of Camel Milk Exosomes

CMEs were prepared as follows: CM was collected from healthy Bactrian camels during the mid-lactation period in Ejina Banner, Inner Mongolia, and immediately stored in ice for further use. CMEs were isolated from the CM via ultra-centrifugation. Specifically, the CM was centrifuged at 8000× *g* for 30 min at 4 °C to discard the upper fat layer, casein micelles, and other cellular debris. The obtained supernatant was then centrifuged at 13,000× *g* for 1 h at 4 °C to remove the residual lipid droplets and cell fragments. Finally, the clarified skimmed supernatant was subjected to ultra-centrifugation at 120,000× *g* for 120 min at 4 °C. The CME precipitate was collected, suspended in PBS, and finally passed through a 0.22 μm sterile filter membrane for further use. The total CME proteins were quantified with a BCA kit (Glpbio Technology, Montclair, CA, USA) [19].

### 2.2. Identification of CMEs

After being fixed in 2.5% glutaraldehyde for 1 h at 20 °C and subsequently stained with 2% phosphotungstic acid, CME morphology was examined using transmission electron microscopy (TEM, Hitachi, Tokyo, Japan). CME size was analyzed by nanoparticle tracking analysis (NTA) using ZetaView (Particle Metrix, Meerbusch, Germany). The expression of exosome-specific surface markers, namely, CD81 and TSG101, was confirmed using Western blotting.

### 2.3. CME Labeling and Cellular Uptake

CMEs were labeled with fluorescent dyes using a kit (Sigma, St. Louis, MI, USA), following a previously established protocol [22]. The labeled CMEs were washed and centrifuged at 100,000× *g* for 1 h. Subsequently, the H9c2 cells were incubated with resuspended CMEs for 6 h to estimate the cellular uptake. The nuclei was stained with DAPI (Beyotime Biotechnology, Shanghai, China). Finally, the images were obtained with a confocal microscope (Nikon A1HD2, Tokyo, Japan).

### 2.4. Animals and Experiments

Adult male C57BL/6 J mice (20 ± 2 g) aged 6–8 weeks were obtained from Huafukang Biotechnology Co., Ltd. (Beijing, China, SPF certificate SCXK [JING] 2019-0008) and housed at a temperature of 24~26 °C. All animal procedures received the approval from the Animal Ethics Committee of Inner Mongolia Agricultural University (approval no. NND2023110). The mice were numbered according to their body weight and randomly divided into a control group, a Dox group, and groups treated with different concentrations of CMEs using random numbers generated by SPSS 26.0 software. The mice in the Dox and CME groups were injected with Dox (4 mg/kg/day) via intraperitoneal injection once every 4 days for 3 weeks, following a previous report [23]. The mice in the control group received an equivalent volume of saline. Additionally, the mice in the CME-treated groups received 0.625, 1.25, and 2.5 mg/kg/day of CMEs intraperitoneally every day for 3 weeks. Body weight and food intake were recorded weekly. All the mice were sacrificed via intraperitoneal injection of tribromoethanol, and the blood and cardiac tissue were obtained 24 h after the final administration of CMEs.

### 2.5. Echocardiography

Mice were placed under isoflurane anesthesia for the echocardiography experiment, which was conducted by Vevo 2100 (FUJIFILM VisualSonics Inc., Toronto, ON, Canada). Multiple cardiac performance parameters were measured to estimate cardiac function.

### 2.6. Serum Biochemical and Histological Analysis

Whole blood samples were collected from the orbital venous plexuses of the mice and kept at room temperature for 30 min, followed by centrifugation at 3000× *g* for 15 min at 4 °C. The harvested serum was stored at −80 °C for subsequent analysis and used to measure the levels of creatine kinase-MB (CK-MB, mlbio, Shanghai, China), N-terminal pro-B-type natriuretic peptide (NT-ProBNP, ml003242B, mlbio), cardiac troponin T (cTnT, ml231456, mlbio), lactate dehydrogenase (LDH, Nanjing Jiancheng, Nanjing, China), and atrial natriuretic peptide (ANP, ml105994, mlbio) via commercial assay kits using an Epoch microplate reader (BioTek Instroments Inc., Winooski, VT, USA).

For histological analysis, the cardiac tissues were fixed with 4% paraformaldehyde, dehydrated with ethanol, and embedded in paraffin. Finally, the embedded tissues were subsequently sectioned for 4 μm to perform hematoxylin and eosin staining (HE) and Masson staining using a microscope (Nikon, Tokyo, Japan).

### 2.7. Cell Culture

The rat cardiomyocytes H9c2 cells were maintained in DMEM (Gibco, Thermo Fisher Scientific, Walthan, MA, USA), 10% FBS (Excell Bio, Suzhou, China), and 1% penicillin–streptomycin (Solarbio, Beijing, China). The human breast carcinoma MCF7 cells and human lung cancer A549 cells were maintained in RPMI 1640 (C11875500BT, Gibco), 10% FBS, and 1% penicillin–streptomycin.

### 2.8. Cell Treatment and Cell Viability Experiment

The cells were treated with 1.56 μg/mL~200 μg/mL of CMEs for 24 h to measure cell viability via MTT methods. The cells, including H9c2 cells, MCF7 cells, and A549 cells, were treated with 1.56 μg/mL~200 μg/mL of CMEs and 2 μM of Dox for 24 h to investigate the cytotoxicity with MTT method.

### 2.9. DCFH-DA Fluorescent and Mitochondria Membrane Potential Measurements

The H9c2 cells were stained with the DCFH-DA fluorescent probe (Beyotime Biotechnology, Shanghai, China) to estimate the ROS production according to previous reports [23]. The mitochondrial membrane potential of the H9c2 cells and apoptosis cardiomyocyte were determined using a kit (C1071S, Beyotime) according to previously reported instructions [24].

### 2.10. Transcriptomic Analysis

After RNA extraction, RNA processing, including quality assessment, library preparation, and high-throughput sequencing, was performed by Wancheng Jingwei Gene Technology Co., Ltd. (Beijing, China; https://www.10kgenomics.com/) on an Illumina HiSeq 2500 platform. Differential expression analysis of the different groups was conducted using the DESeq2 package (v1.34.0), with significantly differentially expressed genes (DEGs) defined by a |fold change| ≥ 2 and an adjusted *p*-value < 0.05. For functional interpretation of the DEGs, Gene Ontology (GO, https://geneontology.org/) enrichment analysis, including biological processes (BPs), cellular components (CCs), and molecular functions (MFs), and Kyoto Encyclopedia of Genes and Genomes (KEGG, http://www.kegg.jp/) pathway enrichment analysis were performed using the clusterProfiler R package (v4.2.2).

### 2.11. Quantitative Real-Time PCR (QT-PCR)

Total RNA was extracted from H9c2 cells and myocardial tissue using a TRIzol reagent (Invitrogen, Carlsbad, CA, USA). The concentration and purity (A260/A280) of RNA were determined via spectrophotometry. Qualified RNA was reverse-transcribed into cDNA using a reverse transcription kit (Invitrogen, Carlsbad, CA, USA). QT-PCR was performed with a SYBR-Green qPCR master mix (Penzberg, Bavaria, Germany) on a real-time PCR detection system (Roche LightCycler 480 II, Penzberg, Bavaria, Germany). GAPDH was used as the internal reference gene. Relative mRNA expression levels were calculated according to the 2^(−∆∆Ct)^ method. The specific primer sequences used in this study are presented in Table 1.

### 2.12. Western Blotting (WB)

Protein lysates from H9c2 cells and cardiac tissues were separated by SDS-PAGE and transferred onto PVDF membranes. The proteins were first incubated with their respective primary antibodies (listed in Table 2) overnight at 4 °C, and then incubated with the horseradish peroxidase-conjugated secondary antibody (ABclonal, Wuhan, China) for 1 h at room temperature. WB bands were visualized using an ultra-high-sensitivity ECL kit (Glpbio Technology, Montclair, CA, USA).

### 2.13. Statistical Analysis

The data, expressed as mean ± SEM, were analyzed using GraphPad Prism 9. The Shapiro–Wilk test was used to evaluate data distribution and normality, and Levene’s test was applied to detect homogeneity of variance before statistical comparison. When data met normal distribution and equal variance assumptions, one-way ANOVA followed by Tukey’s test was used for multi-group comparisons. If data did not meet normality or homoscedasticity, a non-parametric Kruskal–Wallis test with Dunn’s post hoc test was adopted. *p* < 0.05 was considered statistically significant. All experiments were independently repeated at least three times (biological replicates). For Western blot quantitative result, *n* = 3 refers to three independent biological replicates. Each biological replicate also contained 2–3 technical replicates.

## 3. Results

### 3.1. CME Characterization

**Figure 1:**
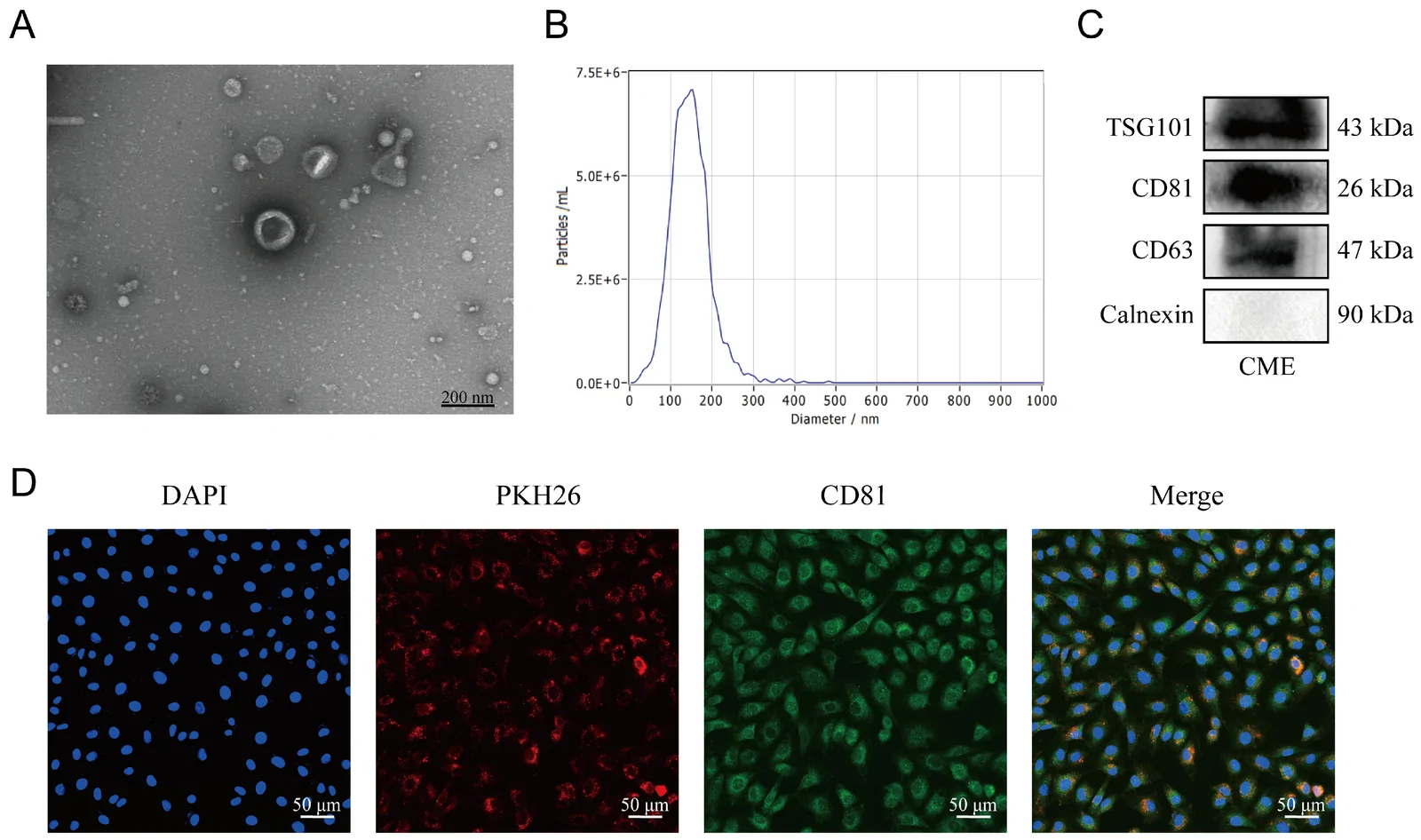
Identification of CME: (**A**) TEM image of isolated CMEs; (**B**) size distribution of CMEs; (**C**) WB bands of TSG101, CD81, CD63, and Calnexin; (**D**) co-localization of PKH26-labeled CMEs and CD81 in H9c2 cells.

The TEM analysis revealed that the morphology of the isolated CMEs was round (Figure 1A). What is more, the NTA revealed that the CME concentration was 7.8 × 10^12^ particles/mL, which has an average size of 146.9 ± 48.7 nm (Figure 1B). Finally, the WB bands of TSG101, CD81, CD63, and Calnexin further confirmed the identity of the CMEs (Figure 1C). We further explored the cellular uptake of the CMEs in H9c2 cells. The images in Figure 1D show that the red fluorescent-labeled CMEs were detected in cytoplasm and co-localized with CD81, which indicates that CMEs can be internalized in H9c2 cells.

### 3.2. CMEs Affected Cell Viability

**Figure 2:**
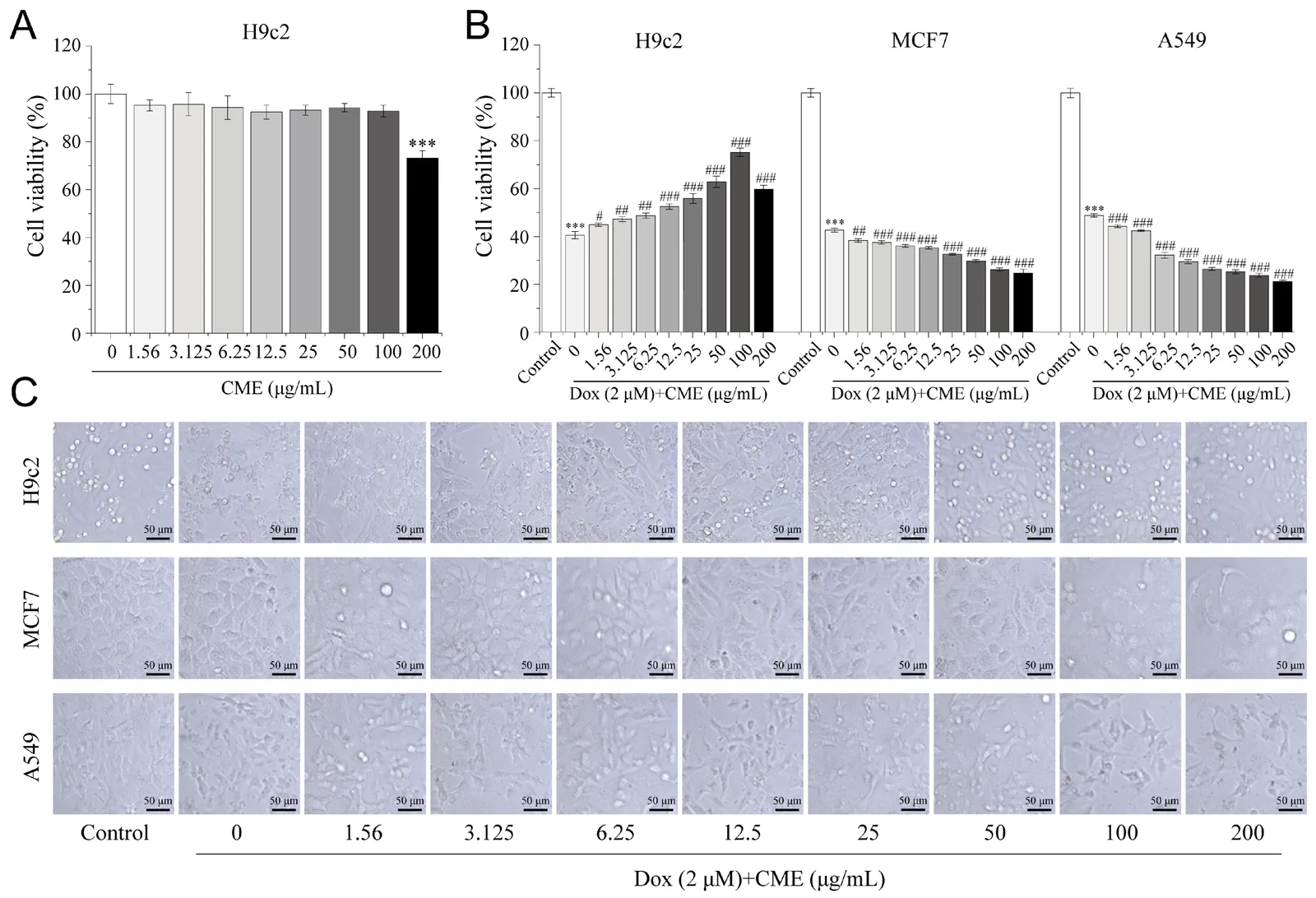
Cell viability of H9c2, MCF7, and A549 cells under co-treatment of Dox and CME: (**A**) H9c2 cell viability under 0~200 uM CME treatment (*n* = 6); (**B**) H9c2 cell, MCF7, and A549 cells viability under 0 μg/mL~200 μg/mL CME and 2 μM Dox treatment (*n* = 6); (**C**) cell morphology of H9c2, MCF7, and A549 cells. All the data are mean ± SEM; *** *p* < 0.001 vs. control, ^#^ *p* < 0.05 vs. 0 μM CME, ^##^ *p* < 0.01 vs. 0 μM CME, and ^###^ *p* < 0.001 vs. 0 μM CME.

Considering the protective effect of exosomes on DIC mice reported in previous studies [25], we aimed to examine whether the CMEs exerted protective effects against DIC. First, Figure 2A indicates that 0~100 μg/mL of the CMEs had no toxicity on H9c2 cell. We further explored the protective effect of the CMEs on DIC in vivo and in vitro. We applied 2 μM of Dox to induce the H9c2 injury model presented in a previous report [23] and estimated the protective effect of the CMEs against DIC in vitro. The results showed that 1.56~100 μg/mL of the CMEs obviously increased the H9c2 cell viability in the Dox-treated group (Figure 2B,C). Furthermore, we checked the viability of MCF-7 and A549 cells in the presence of the CMEs and Dox; the results indicated that the CMEs had a synergistic anti-tumor effect in Dox treatment (Figure 2B,C).

### 3.3. CMEs Improved the Cardiac Function of DIC Mice

**Figure 3:**
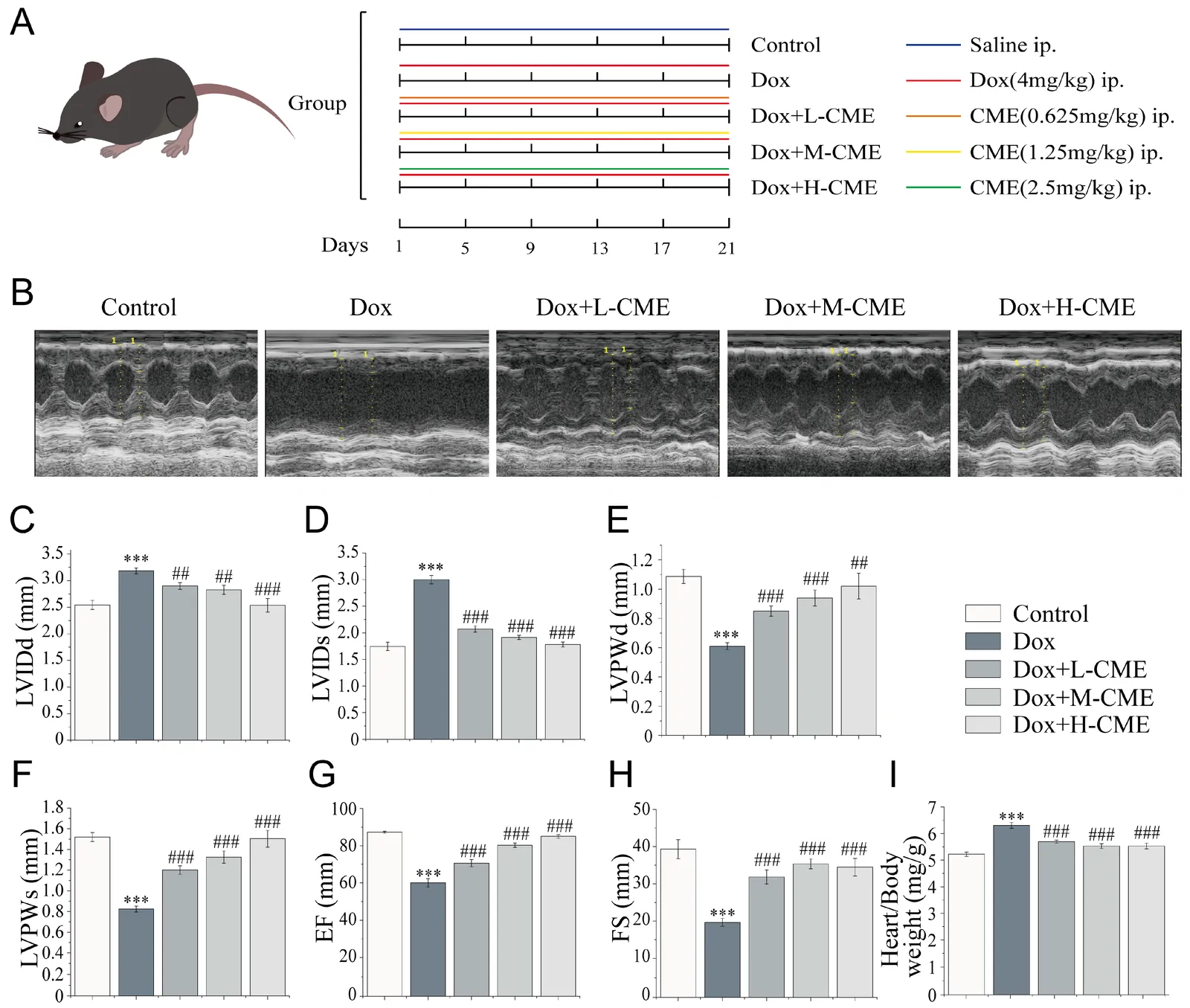
Cardiac function measured by echocardiography: (**A**) schematic representation of animal experimental protocol; (**B**) representative echocardiography images; 1, measurement cursors. (**C**–**H**) quantitative analysis of echocardiography indicators (*n* = 6); (**I**) heart–body weight ratio (*n* = 6). All the data are mean ± SEM; *** *p* < 0.001 vs. Control, ^##^ *p* < 0.01 vs. Dox, and ^###^ *p* < 0.001 vs. Dox.

Having confirmed the cytoprotective effect of the CMEs against DIC in vitro, we further explored their protective effect against DIC in mice. We first evaluated the impact of the CMEs on the cardiac function of mice in vivo. The animal experiment was conducted as shown in Figure 3A. We estimated the cardiac function of the mice via an echocardiography experiment, with the results implying that the CMEs improved the cardiac function of DIC mice by regulating left ventricular posterior wall thickness at end-diastole (LVPWd), left ventricular posterior wall thickness at end-systole (LVPWs), left ventricular internal dimension at end-systole (LVIDs), left ventricular internal dimension at end-diastole (LVIDd), fractional shortening (FS), and ejection fraction (EF) (Figure 3B–H). In addition, we found that the CMEs could also significantly increase the heart–body weight ratio of DIC mice (Figure 3I).

### 3.4. CMEs Improved Cardiac Injury Indicators and Pathology of DIC Mice

**Figure 4:**
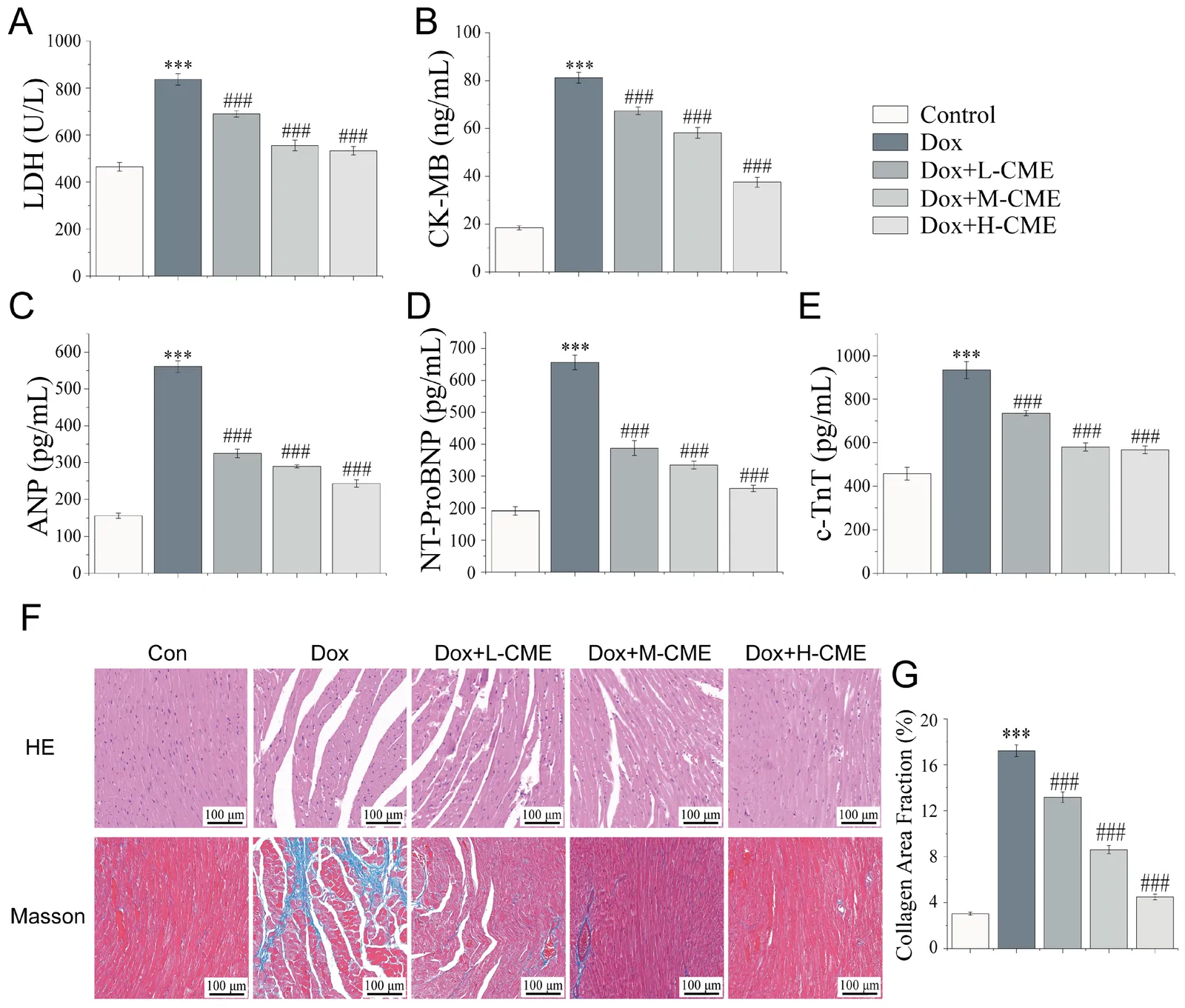
CMEs improved cardiac injury markers of DIC mice: (**A**–**E**) quantitative analysis of cardiac injury markers (*n* = 6); (**F**) representative images of HE and Masson staining. Red indicates cardiomyocytes, and blue represents collagen fibers; (**G**) quantification of Masson staining (*n* = 6). All the data are mean ± SEM; *** *p* < 0.001 vs. Control, ^###^ *p* < 0.001 vs. Dox.

We further analyzed the cardiac injury indicators of mice. The results in Figure 4A–E indicate that the CMEs significantly decreased the cTnT, LDH, ANP, CK-MB, and NT-ProBNP levels of Dox-treated mice, which further illustrates that the CMEs alleviated cardiac injury in DIC mice. Meanwhile, the HE and Masson images also implied that the CMEs can alleviate cardiac pathological injury due to DIC mice (Figure 4F,G).

### 3.5. CMEs Affected the Transcriptome Result of DIC Mice

**Figure 5:**
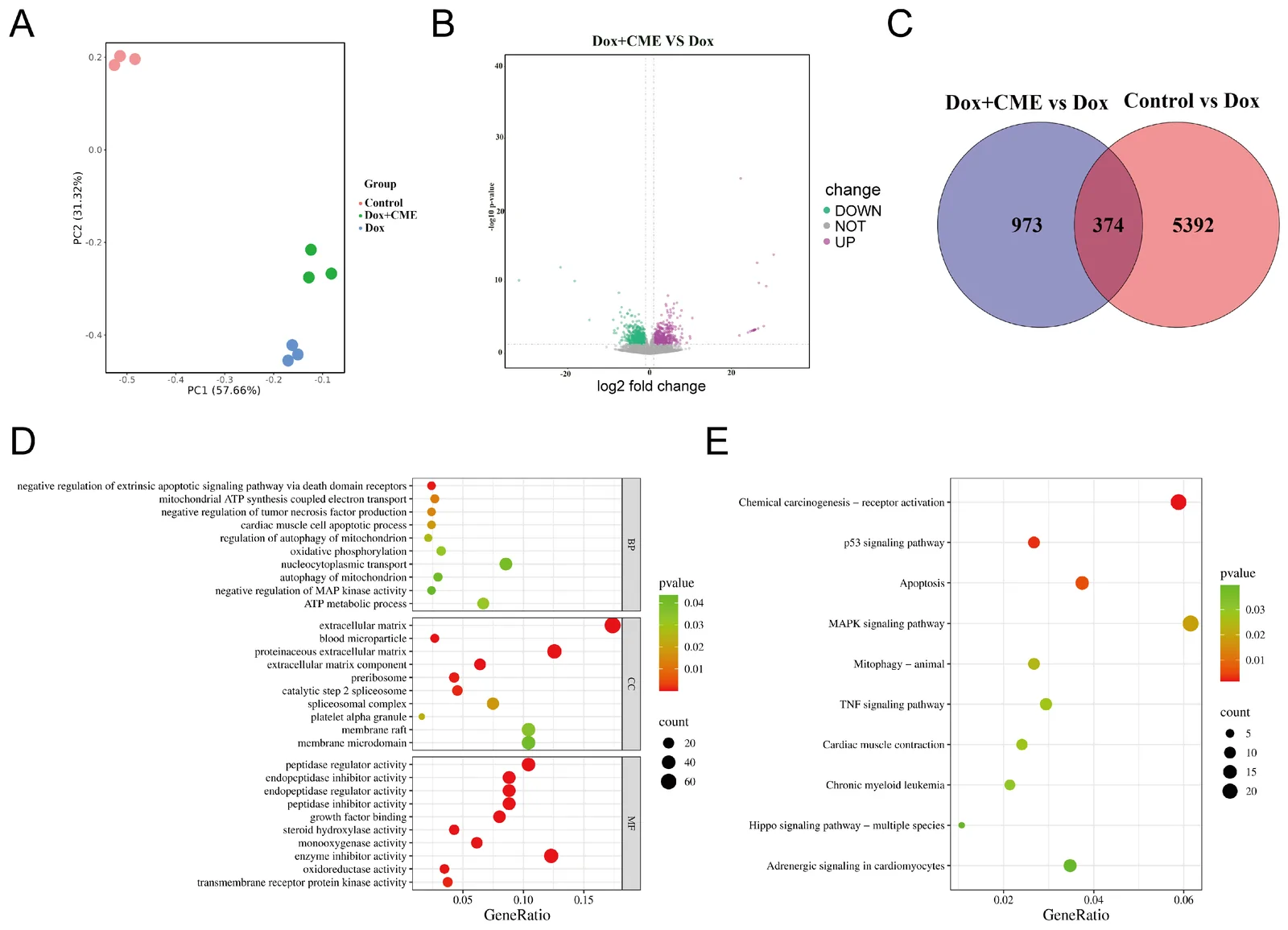
Transcriptome analysis of CMEs on DIC mice: (**A**) PCA analysis of transcriptome data; (**B**) volcano plots of DEGs; (**C**) Venn diagrams of 374 DEGs; (**D**) bubble chart of the top 10 of the enriched BPs, CCs, and MFs; (**E**) bar chart of the top 10 of KEGG pathway analysis.

The PCA analysis in Figure 5A demonstrates the significant differences in transcriptomic analysis among the control, Dox, and Dox + CME groups, which indicated that the RNA-seq data was suitable for the subsequent DEGs screening and functional enrichment analysis. To investigate the underlying mechanisms of the CMEs’ cardiac protective effects, the transcriptome sequencing on cardiac tissue was performed. The results in Figure 5B show that there were 640 DEGs up-regulated and 707 DEGs down-regulated in the Dox + CME group compared to the Dox group. Notably, 374 DEGs were identified when comparing the control vs. Dox and Dox vs. Dox + CME groups (Figure 5C). DEGs enriched in BPs, CCs, and MFs are presented in Figure 5D. Meanwhile, KEGG pathway analysis further highlighted that the targets were mainly associated with the p53, PI3K-Akt, MAPK, and TNF signaling pathways (Figure 5E). The transcriptome analysis results suggest that CMEs exert cardioprotective effects by modulating multiple molecular pathways.

### 3.6. CMEs Regulated Apoptosis and Autophagy

**Figure 6:**
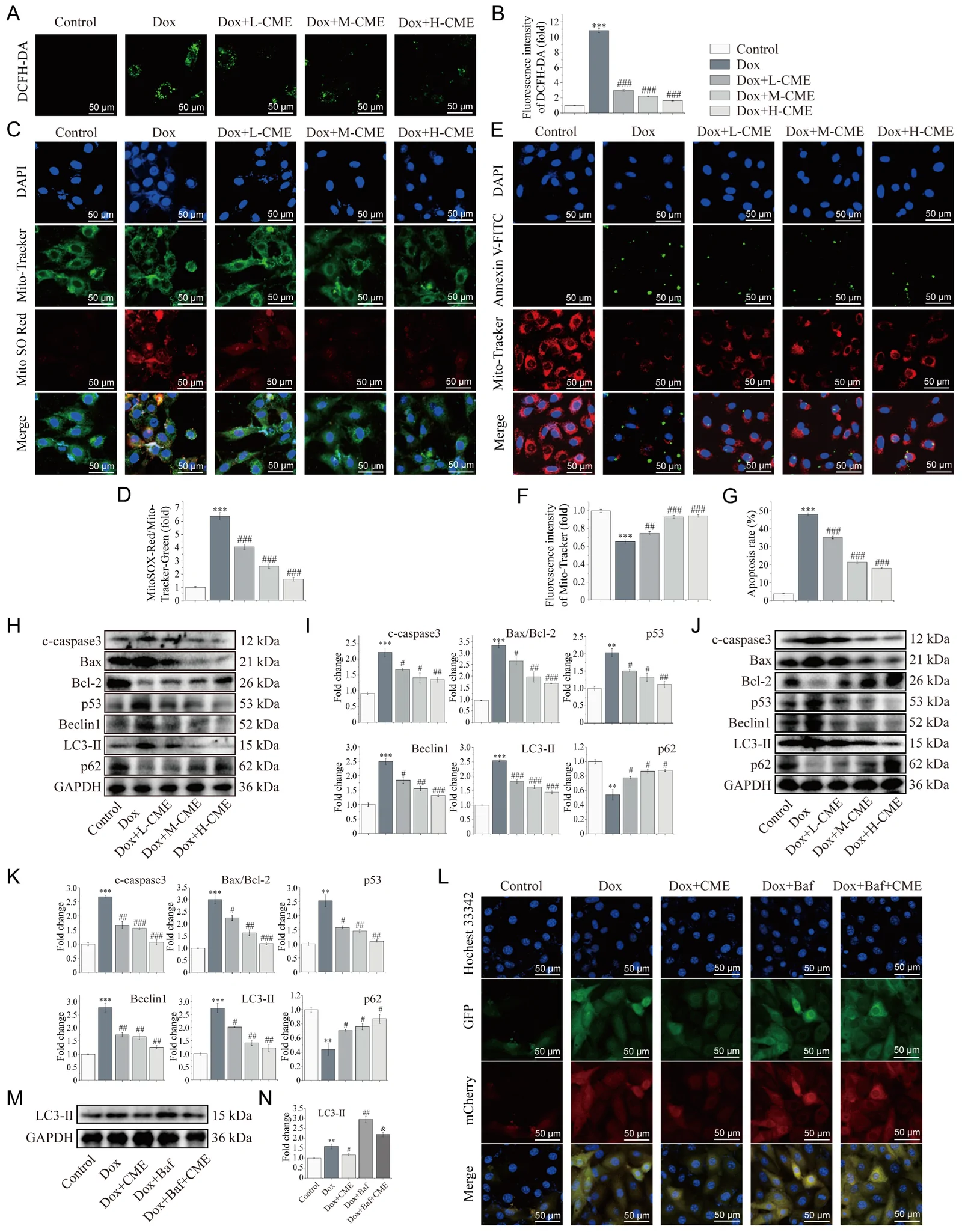
CMEs attenuated DIC by regulating apoptosis and autophagy: (**A**) representative images of DCFH-DA fluorescence staining; (**B**) fluorescence intensity of DCFH-DA (*n* = 6); (**C**) representative images of MitoSOX fluorescence staining; (**D**) fluorescence intensity of MitoSOX (*n* = 6); (**E**) representative images of annexin V-FITC and Mito-Tracker fluorescence staining; (**F**) fluorescence intensity of Mito-Tracker (*n* = 6); (**G**) apoptosis rate of H9c2 cells (*n* = 6); (**H**) representative WB images in DIC mice; (**I**) quantitative analysis of WB bands in DIC mice (*n* = 3); (**J**) representative WB images in H9c2 cells; (**K**) quantitative analysis of WB bands in H9c2 cells (*n* = 3); (**L**) autophagic flux detected by mCherry-GFP-LC3 in H9c2 cells; (**M**) representative WB image of LC3Ⅱ in H9c2 cells; (**N**) quantitative analysis of WB image of LC3Ⅱ in H9c2 cells (*n* = 3). All the data are mean ± SEM; ** *p* < 0.01 vs. Control, *** *p* < 0.001 vs. Control, ^#^ *p* < 0.05 vs. Dox, ^##^ *p* < 0.01 vs. Dox, ^###^ *p* < 0.001 vs. Dox, ^&^ *p* < 0.05 vs. Dox + Baf.

It has been reported that ROS production and mitochondrial membrane potential are closely associated with apoptosis and autophagy in the DIC models [23], which are assessed in Figure 6. The results in Figure 6A–D indicate that the CMEs down-regulated the increased ROS and MitoSOX levels. We further investigated cell apoptosis in vitro. The apoptosis of Dox-treated H9c2 cells clearly increased, which was attenuated by the CME treatment (Figure 6E–G). CMEs could also improve the damaged mitochondria in Dox-treated H9c2 cells (Figure 6E–G). Additionally, WB results in vivo and in vitro reveal that CMEs can decrease c-caspase3 and Bax protein levels, while increasing the Bcl-2 protein level in the Dox-treated group (Figure 6H–K and Supplementary Materials). Furthermore, the CME treatment modulated autophagy by decreasing LC3-II and Beclin-1 protein levels, while increasing the p62 protein in Dox-treated H9c2 cells (Figure 6H–K and Supplementary Materials). To further explore the interaction between CMEs and autophagy, autophagy flux was measured in H9c2 cells [26]. As shown in Figure 6L, autophagic flux was evaluated using the Ad-mCherry-GFP-LC3B adenovirus kit (Beyotime Biotechnology, Shanghai, China). In control H9c2 cells, few LC3 puncta were observed. Dox treatment markedly increased the number of yellow autophagosome puncta, with limited pure-red autolysosome puncta, which indicates an accumulation of autophagosomes and impaired autophagic degradation. Compared with the Dox group, the CME-treated group exhibited reduced yellow puncta, suggesting that the CMEs alleviated autophagosome accumulation triggered by Dox. In the presence of Bafilomycin A1 (Baf, a lysosomal degradation inhibitor), abundant yellow puncta were accumulated in both Dox + Baf and Dox + Baf + CME groups. Upon the inhibition of lysosomal under the Baf treatment, massive yellow autophagosome puncta accumulated in both the Dox + Baf and Dox + Baf + CME groups, while the CMEs failed to reduce autophagosome accumulation when lysosomal degradation was blocked. The WB results of LC3 in Figure 6M,N and Supplementary Materials could further confirm the results of Figure 6L. The results indicate that the protective effect of CMEs on autophagic flux requires functional lysosomal activity. These results collectively indicate that the CMEs exerted a cardiac protective effect against DIC by modulating apoptosis and autophagy, thereby maintaining mitochondrial stability and reducing oxidative stress in the DIC models.

### 3.7. CMEs Regulated NF-κB p65 and MAPK Pathways

**Figure 7:**
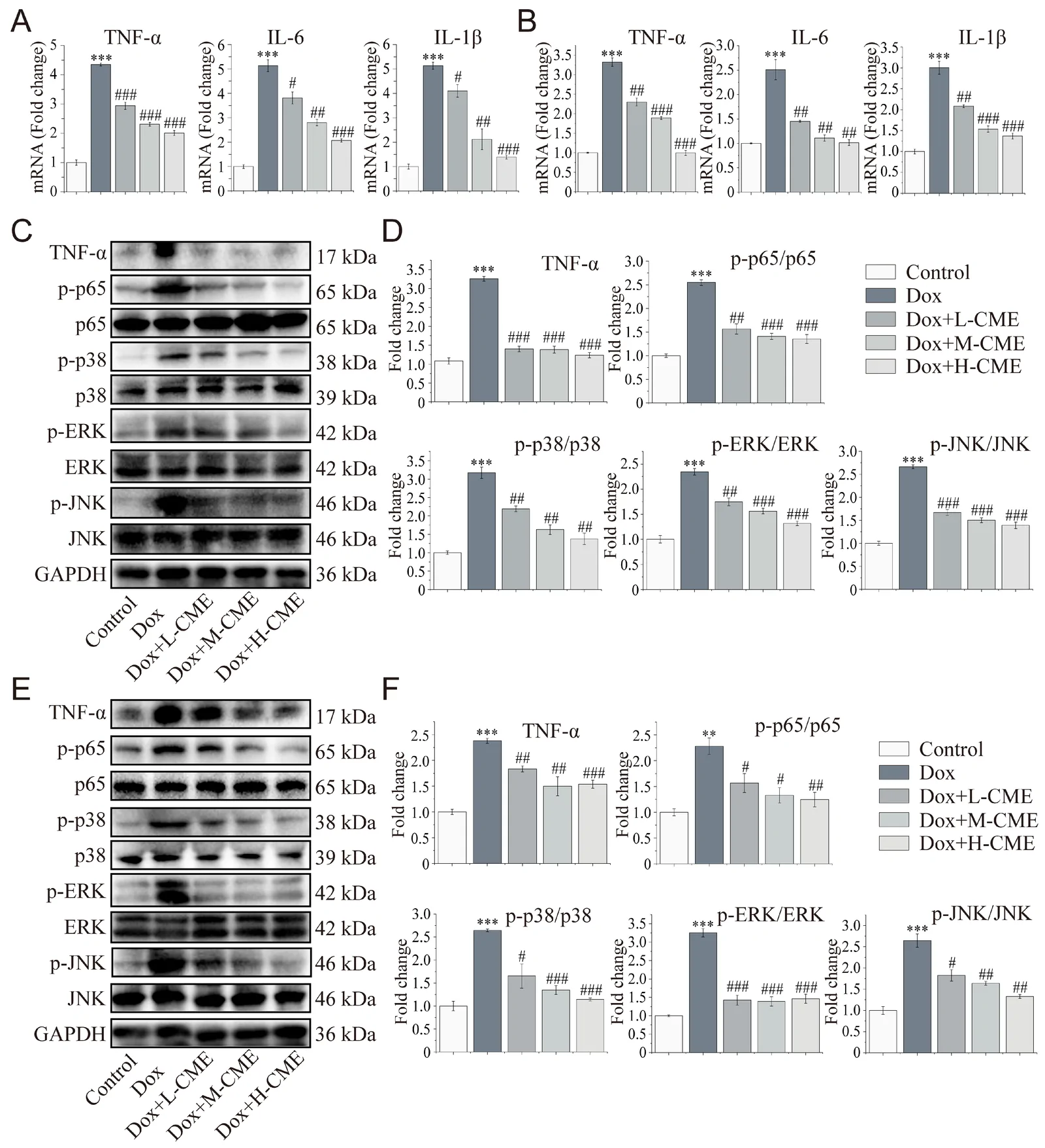
CMEs regulated NF-κB p65 and MAPK pathways: (**A**) the TNF-α, IL-6, and IL-1β levels in DIC mice measured by QT-PCR (*n* = 3); (**B**) the TNF-α, IL-6, and IL-1β levels in H9c2 cells measured by QT-PCR (*n* = 3); (**C**) representative WB images in DIC mice; (**D**) quantitative analysis of WB bands in DIC mice (*n* = 3); (**E**) representative WB images in H9c2 cells; (**F**) quantitative analysis of WB bands in H9c2 cells (*n* = 3). All the data are mean ± SEM; ** *p* < 0.01 vs. Control, *** *p* < 0.001 vs. Control, ^#^ *p* < 0.05 vs. Dox, ^##^ *p* < 0.01 vs. Dox, ^###^ *p* < 0.001 vs. Dox.

Previous reports have shown that the NF-κB p65 and MAPK pathways are both associated with the regulation of apoptosis and autophagy in the presence of Dox [27]. Moreover, these two pathways have been implicated in DIC development and treatment [8]. To further investigate the underlying mechanisms, we examined the effects of the CMEs on NF-κB p65 and MAPK pathways. At first, we estimated the TNF-α, IL-6, and IL-1β levels via QT-PCR in vivo and in vitro. The results indicate that the CMEs could alleviate the increased TNF-α, IL-6, and IL-1β levels in the Dox groups (Figure 7A,B). Figure 7C,D and Supplementary Materials shows that Dox addition up-regulated p-NF-κB p65 and p-IκBα expression, as well as TNF-α expression, while the CME treatment markedly attenuated these effects. Furthermore, the CMEs also suppressed the Dox-induced activation of MAPK signaling, as evidenced by the reduced phosphorylation of JNK, ERK1/2, and p38 (Figure 7E,F and Supplementary Materials). These results suggested that the CMEs exerted their cardiac protective effect, at least in part, by inhibiting the NF-κB p65 and MAPK pathways in vivo and in vitro. On the whole, this study provided evidence that CMEs are promising natural bioactive agents for mitigating DIC, offering a novel therapeutic strategy for the prevention of chemotherapy-related cardiac injury.

## 4. Discussion

As a widely used chemotherapy drug, Dox application is severely restrained by its dose-dependent cardiotoxicity, which is characterized by irreversible cardiomyopathy and heart failure [28]. To date, the effective agents for DIC are still limited; dexrazoxane is the only FDA-approved drug, and it possesses a number of side effects [10]. Consequently, there is an urgent need to identify novel, safe, and effective drugs for DIC treatment. In recent years, natural bio-active products and extracellular vesicles have received more attention in the field of cardiovascular disease treatment [29]. CM, which has nutritional and medicinal properties, has been identified as a rich source of exosomes with both stability and bio-activity [30]. However, the specific roles of CMEs in DIC treatment have not previously been investigated. After isolation, the CME characterizations were confirmed through TEM, NTA, and WB, with the results being consistent with the reported exosomes criteria [17]. Additionally, the observed spherical morphology and size distribution of CMEs agree with those of previous reports concerning exosomes obtained from milk [31].

Our study is the first to systematically investigate the heart-protective effect of CMEs in DIC mice and Dox-treated H9c2 cells. The findings in our research demonstrate that the CMEs effectively mitigated DIC both in vivo and in vitro through oxidative stress inhibition, apoptosis inhibition, and autophagy regulation via the NF-κB p65 and MAPK pathways. The cardiac protection effects of CMEs against DIC did not compromise the anti-tumor efficacy of Dox, which suggested to us that CMEs did not interfere with the inhibition of Dox in tumor cells with respect to cardiac protection. Furthermore, a synergistic anti-tumor effect was also observed, which is an obvious advantage of CME. The distinct responses of H9c2 and tumor cells to CME co-treatment with Dox require further exploration, including cellular uptake and subcellular localization verification.

The in vivo results showed that CME treatment significantly improved the cardiac function of DIC mice, as evidenced by the improvement in echocardiography parameters (such as EF, FS, LVIDd, and LVIDs) and cardiac injury markers (namely, LDH, NT-ProBNP, cTnT, CK-MB, and ANP). HE and Masson staining further confirmed the protective effect of CMEs in DIC mice. These findings are consistent with reports that exosomes can exert cardiac protection effects [32,33].

It has been reported that oxidative stress is among the causes of DIC, which subsequently triggers mitochondrial dysfunction and programmed cell death [34]. Our results demonstrate that CMEs significantly reduced intracellular ROS production and alleviated mitochondrial damage in Dox-treated H9c2 cells. The anti-oxidative effect is associated with apoptosis to a great extent, which is downstream of oxidative stress [35]. CME treatment effectively inhibited H9c2 cells apoptosis, down-regulated Bax and c-caspase 3 expression, and up-regulated Bcl-2 expression in DIC mice and H9c2 cells. Our research is in line with previous studies demonstrating that CM has an antioxidant effect and extends their findings to CMEs [16]. It has been reported that autophagy plays a dual role in DIC development and treatment, and its dysregulation contributes to cell death via several mechanisms [8]. Our data indicate that CMEs regulated autophagy in DIC models, as evidenced by the decreased LC3II and Beclin-1 expression, as well as the increase in p62 expression. These results suggest that CMEs protect against Dox-induced injury of cardiomyocytes by clearing the damaged mitochondria and attenuating oxidative stress, thereby promoting cell survival. To further explore the molecular mechanisms, we performed transcriptome analysis, which revealed that CMEs modulate several signaling pathways, including the TNFα and MAPK pathways. TNF-α is a vital inflammatory cytokine up-regulated in the DIC model, which can activate the NF-κB pathway, leading to pro-apoptotic genes transcription [8]. The WB results confirmed that CMEs suppress the increase in TNF-α and p-NF-κB p65 expression caused by Dox, indicating inhibition of the NF-κB p65 pathway in the CME-treated group. Additionally, the MAPK family is critically involved in DIC development [36]. We found that CMEs significantly reduced the phosphorylation of ERK1/2, JNK, and p38 expressions. Finally, by suppressing NF-κB and MAPK singling pathways, CMEs protected DIC by effectively dampening the inflammatory and stress signals, which provided a mechanism for its protective effects. In this research, we systematically clarified the protective mechanism of CMEs against DIC through the modulation of autophagy homeostasis and inflammatory signaling. Unlike conventional cardiac protection agents, which may interfere with the cancer therapy, CMEs display a unique dual regulation mechanism, which greatly improves their clinical applicability. These findings not only reveal a new functional mechanism of CMEs but also provide an effective experimental basis for the development of natural adjuvant therapies based on exosomes to reduce chemotherapy-related cardiac complications. Currently, dexrazoxane remains the only approved agent for preventing DIC [37] in clinical settings. Nevertheless, its clinical application is restricted by obvious drawbacks [38]. In contrast, CMEs can prevent DIC while enhancing chemosensitivity for cancer treatment. Compared with dexrazoxane, CMEs demonstrated no hematological toxicity in the present study, indicating its promising translation prospect as an alternative or adjuvant cardioprotective candidate. However, further pre-clinical safety evaluation is essential before clinical application of CMEs.

This research has a number of limitations. First, although the present study demonstrated that CMEs effectively alleviated Dox-induced cardiac inflammation, abnormal autophagy, and myocardial injury by regulating the NF-κB and MAPK signaling pathways, the specific bioactive components involved in the protection effect, including lipids, proteins, and microRNAs, remain unidentified. Second, the animal experiment conducted in this research was evaluated in the short term; long-term toxicological evaluation, organ bio-safety detection, and immune reaction after continuous CME administration are still absent. Third, only a single Dox concentration (2 μM) was applied in the in vitro experiment. Further experiments with a series of Dox concentrations are required to confirm whether the protective effect of CMEs is dose-dependent. Determining long-term medication safety is essential for the future clinical transformation of cardiac protective agents; therefore, further systematic safety assessments are required before CME application can be approved for DIC patients. Our findings provide strong evidence for CME development as a candidate drug for DIC in clinical settings. Nevertheless, several obstacles limit the clinical translation of CME. The core active components of CMEs remain unclear, and its dosage form requires further optimization. In addition, the safe and effective dosage for human patients has not yet been established. These issues need to be resolved before CMEs can be applied in clinical settings.

## 5. Conclusions

In summary, our research clarified the related protective signaling mechanism of CMEs against DIC. Specifically, CMEs inhibited the over-activated NF-κB/MAPK inflammatory cascade and restored disordered autophagy, which collectively suppressed cardiomyocyte apoptosis and ameliorated myocardial dysfunction. In addition, CMEs exhibited dual activity, providing cardioprotection against DIC and sensitizing tumors to Dox.

## Figures and Tables

**Table 1:** Primer sequences for quantitative QT-PCR.

Gene Name	Forward Prime (5′-3′)	Reverse Prime (3′-5′)
TNF-α	CCACCACGCTCTTCTGTCTACTGAACTT	GTGGGCTACAGGCTTGTCACTCG
IL-6	ACAAAGCCAGAGTCCTTCAGAGAGATACAG	TGAATTGGATGGTCTTGGTCCTTAGCCAC
IL-1β	TGAGAATGACCTGTTCTTTGAAGTTG	GACAGCCCAGGTCAAAGGTTT
GAPDH	GTATGACTCCACTCACGGCAAA	GGTCTCGCTCCTGGAAGATG

**Table 2:** Primary antibodies for Western blotting.

Name	Catalog	Species	Dilutions	Vendor
GAPDH	10494-1-AP	Rabbit	1:5000	Proteintech, Wuhan, China
Bcl-2	12789-1-AP	Rabbit	1:3000	Proteintech, Wuhan, China
Bax	50599-1-AP	Rabbit	1:4000	Proteintech, Wuhan, China
caspase-3	19677-1-AP	Rabbit	1:2000	Proteintech, Wuhan, China
Beclin 1	11306-1-AP	Rabbit	1:2000	Proteintech, Wuhan, China
LC3	18725-1-AP	Rabbit	1:300	Proteintech, Wuhan, China
p62	18420-1-AP	Rabbit	1:3000	Proteintech, Wuhan, China
NF-κB p65	F0006	Rabbit	1:1000	Selleck, Houston, TX, USA
Phospho-NF-κB p65	F0155	Rabbit	1:1000	Selleck, Houston, TX, USA
TNF-α	346654	Rabbit	1:1000	Zenbio, Durham, NC, USA
JNK1/2/3	R22866	Rabbit	1:1000	Zenbio, Durham, NC, USA
Phospho-JNK1/2/3	R381100	Rabbit	1:1000	Zenbio, Durham, NC, USA
Phospho-p38	310091	Rabbit	1:1000	Zenbio, Durham, NC, USA
p38	8690	Rabbit	1:1000	Cell Signaling Technology, Danvers, MA, USA
ERK1/2	343830	Rabbit	1:1000	Zenbio, Danvers, MA, USA
Phospho-ERK1/2	R24245	Rabbit	1:1000	Zenbio, Danvers, MA, USA

## Data Availability

The original contributions presented in this study are included in the article/Supplementary Material. Further inquiries can be directed to the corresponding author.

## Supplementary Materials

The following supporting information can be downloaded at https://www.mdpi.com/article/10.3390/biology15181604/s1, Supplementary File S1: Full-length uncropped Western blot.
