# Supplementary material for: Camel Milk Exosomes Alleviate Doxorubicin-Induced Cardiotoxicity by Regulating Apoptosis and Autophagy via the NF-κB and MAPK Pathways
Source: Biology (Basel). 2026 Sep 11;15(18):1604. doi: 10.3390/biology15181604 (PMC13604618; doi:10.3390/biology15181604)
Supplement: Supplementary file 1 [file biology-15-01604-s001.zip › Supplementary File S1.pdf]

Figure 1.C Western blot bands of CD81, TSG101, CD63 and

Calnexin. CD81:

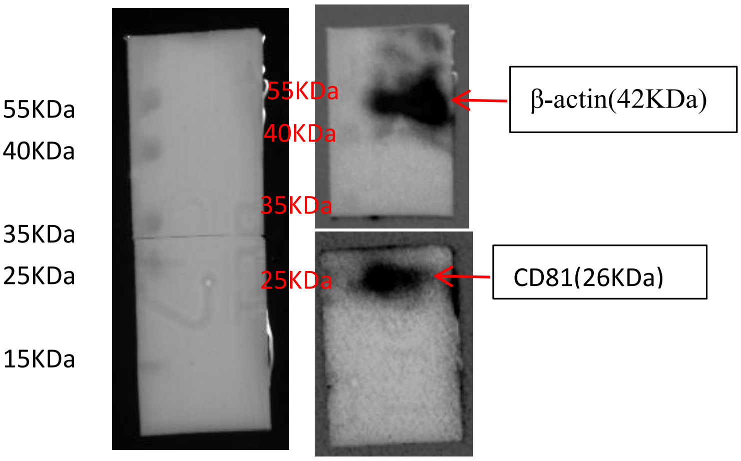

Figure S1. Western blot membrane of CD81 (26 kDa) protein detected with Anti-CD81 (T56400, Abmart) antibody. Gel-separated proteins were transferred to PVDF (0.2 um, millipore). Membranes, incubated with a horseradishperoxidase-conjugated secondary antibody (AS014, ABclonal), were developed with ECL Kit (GK10008, GlpBio). #Weight marker(molecular weight in kDa): SparkJade™ Prestained Protein Ladder, 10 to 180 kDa; catalog number: EC2019.

TSG101:

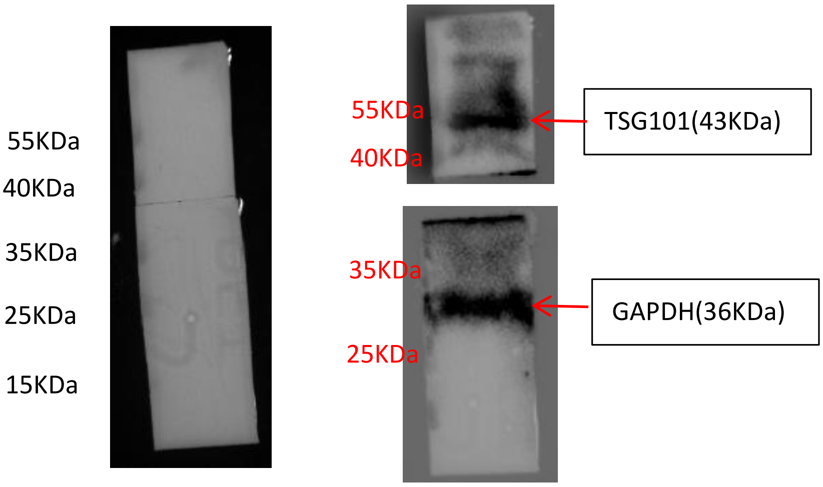

Figure S2. Western blot membrane of TSG101 (43 kDa) protein detected with Anti-TSG101 (T56548, Abmart) antibody. Gel-separated proteins were transferred to PVDF (0.2 um, millipore). Membranes, incubated with a horseradishperoxidase-conjugated secondary antibody (AS014, ABclonal), were developed with ECL Kit (GK10008, GlpBio). #Weight marker(molecular weight in kDa): SparkJade™ Prestained Protein Ladder, 10 to 180 kDa; catalog number: EC2019.

CD63:

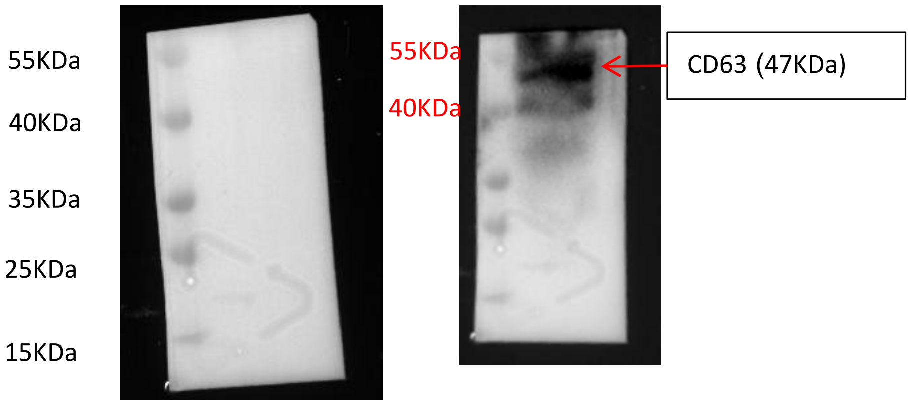

Figure S3. Western blot membrane of CD63 (47 kDa) protein detected with Anti-CD63 (M051014, Abmart) antibody. Gel-separated proteins were transferred to PVDF (0.2 um, millipore). Membranes, incubated with a horseradishperoxidase-conjugated secondary antibody (AS014, ABclonal), were developed with ECL Kit (GK10008, GlpBio). #Weight marker(molecular weight in kDa): SparkJade™ Prestained Protein Ladder, 10 to 180 kDa; catalog number: EC2019.

Calnexin:

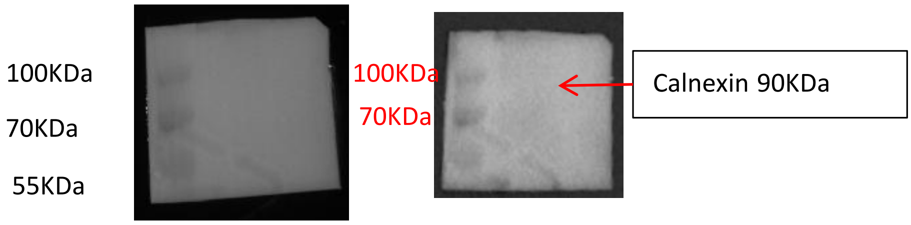

Figure S4. Western blot membrane of Calnexin (90 kDa) protein detected with Anti-Calnexin (TA5362, Abmart) antibody. Gel-separated proteins were transferred to PVDF (0.2 um, millipore). Membranes, incubated with a horseradishperoxidase-conjugated secondary antibody (AS014, ABclonal), were developed with ECL Kit (GK10008, GlpBio). #Weight marker(molecular weight in kDa): SparkJade™ Prestained Protein Ladder, 10 to 180 kDa; catalog number: EC2019.

Figure 6.H Representative WB images in DIC

mice. Cleaved caspase3:

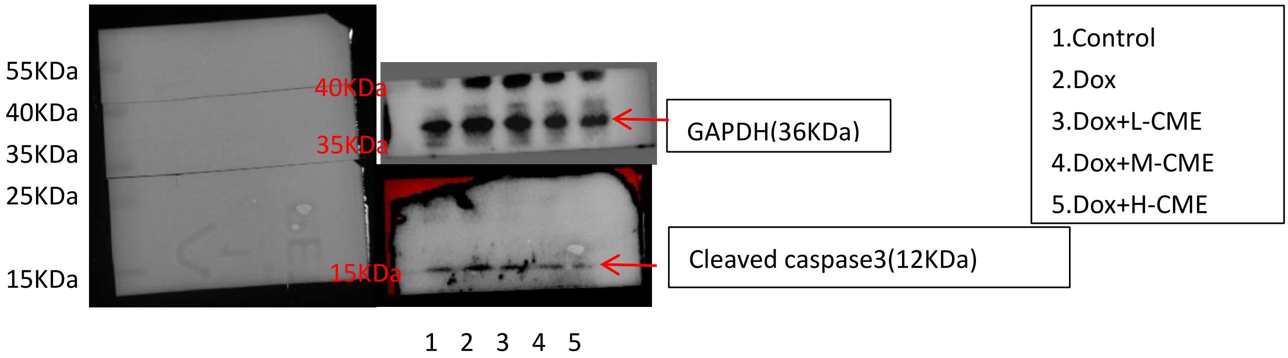

Figure S5. Western blot membrane of Cleaved caspase3 (12 kDa) protein detected with Anti-Cleaved caspase3 (19677-1-AP, Proteintech) antibody. Gel-separated proteins were transferred to PVDF (0.2 um, millipore). Membranes, incubated with a horseradishperoxidase-conjugated secondary antibody (AS014, ABclonal), were developed with ECL Kit (GK10008, GlpBio). #Weight marker(molecular weight in kDa): SparkJade™ Prestained Protein Ladder, 10 to 180 kDa; catalog number: EC2019.

BAX:

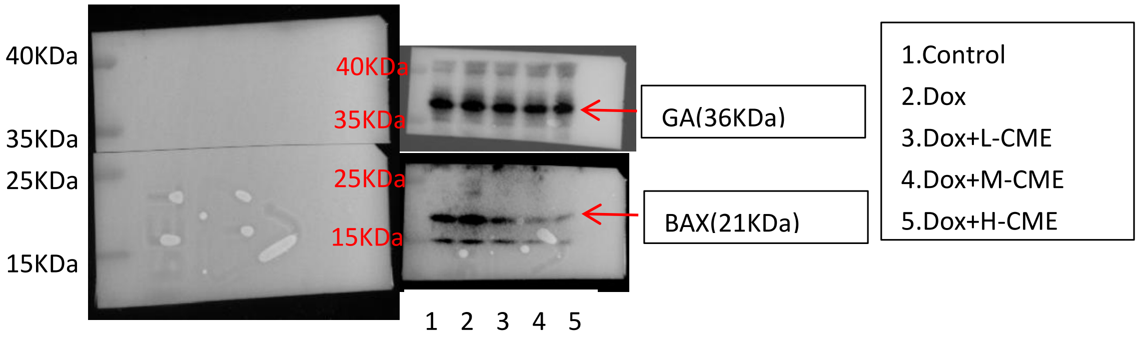

Figure S6. Western blot membrane of BAX (21 kDa) protein detected with Anti-BAX (50599-1-AP, Proteintech) antibody. Gel-separated proteins were transferred to PVDF (0.2 um, millipore). Membranes, incubated with a horseradishperoxidase-conjugated secondary antibody (AS014, ABclonal), were developed with ECL Kit (GK10008, GlpBio). #Weight marker(molecular weight in kDa): SparkJade™ Prestained Protein Ladder, 10 to 180 kDa; catalog number: EC2019.

Bcl-2:

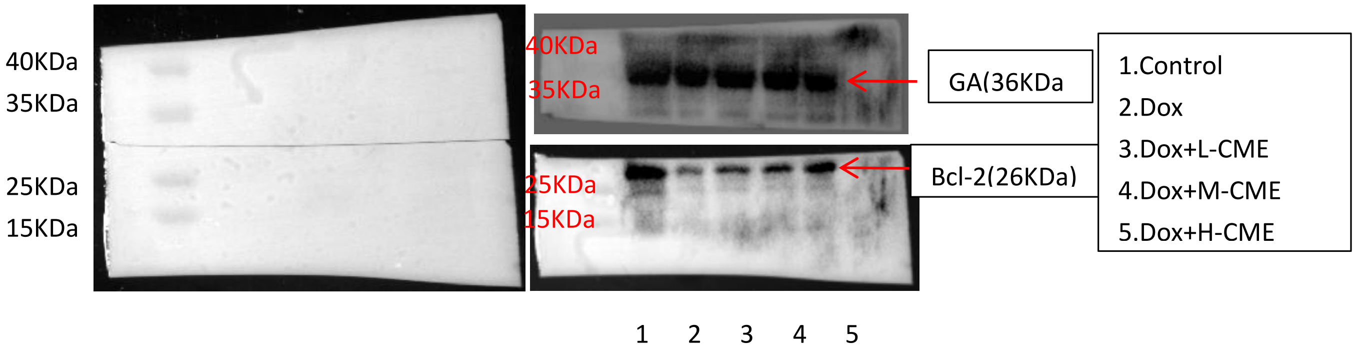

Figure S7. Western blot membrane of Bcl-2 (26 kDa) protein detected with Anti-Bcl-2 (12789-1-AP, Proteintech) antibody. Gel-separated proteins were transferred to PVDF (0.2 um, millipore). Membranes, incubated with a horseradishperoxidase-conjugated secondary antibody (AS014, ABclonal), were developed with ECL Kit (GK10008, GlpBio). #Weight marker(molecular weight in kDa): SparkJade™ Prestained Protein Ladder, 10 to 180 kDa; catalog number: EC2019.

p53:

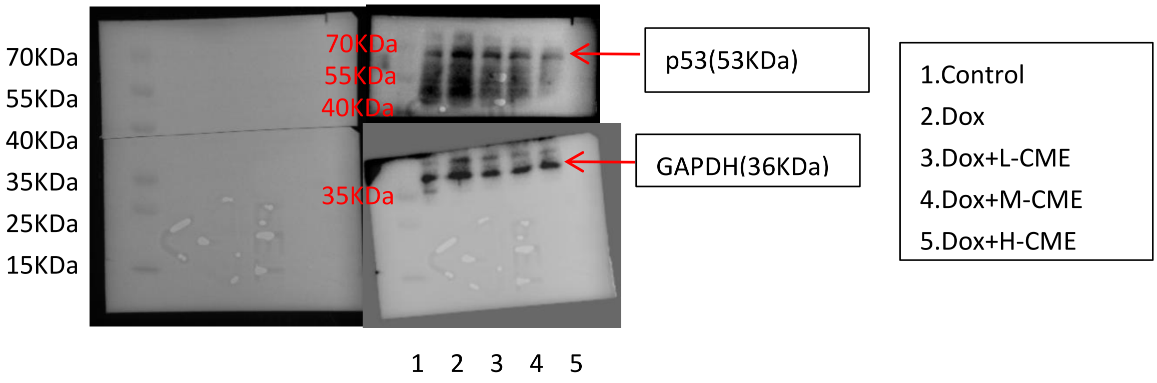

Figure S8. Western blot membrane of p53 (53 kDa) protein detected with Anti-p53 (10442-1-AP, Proteintech) antibody. Gel-separated proteins were transferred to PVDF (0.2 um, millipore). Membranes, incubated with a horseradishperoxidase-conjugated secondary antibody (AS014, ABclonal), were developed with ECL Kit (GK10008, GlpBio). #Weight marker(molecular weight in kDa): SparkJade™ Prestained Protein Ladder, 10 to 180 kDa; catalog number: EC2019.

Beclin-1:

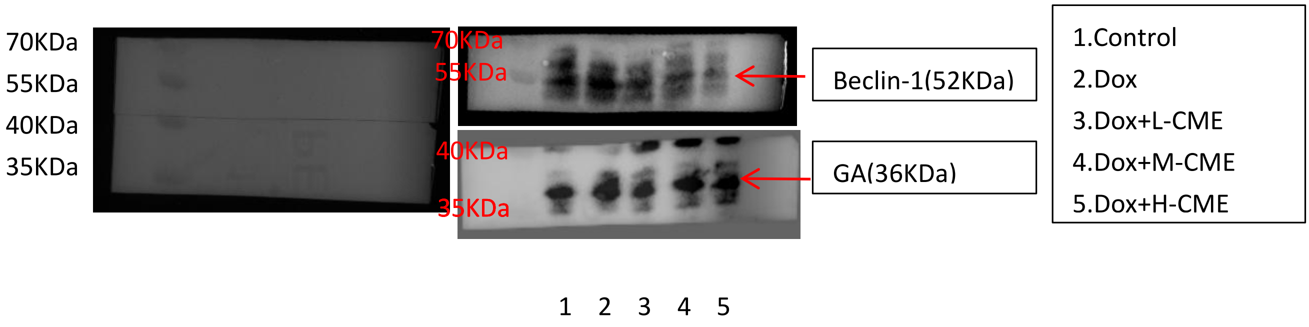

Figure S9. Western blot membrane of Beclin-1 (52 kDa) protein detected with Anti-Beclin-1 (11306-1-AP, Proteintech) antibody. Gel-separated proteins were transferred to PVDF (0.2 um, millipore). Membranes, incubated with a horseradishperoxidase-conjugated secondary antibody (AS014, ABclonal), were developed with ECL Kit (GK10008, GlpBio). #Weight marker(molecular weight in kDa): SparkJade™ Prestained Protein Ladder, 10 to 180 kDa; catalog number: EC2019.

LC3-II:

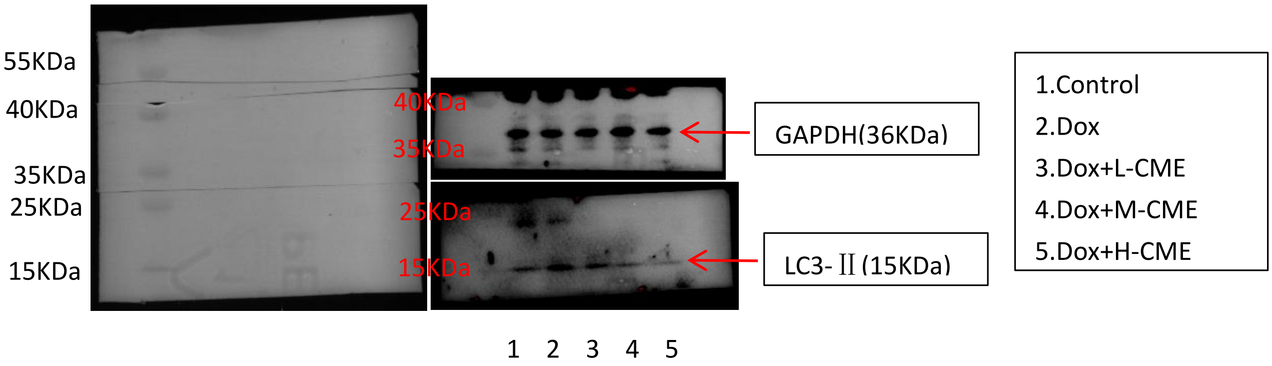

Figure S10 . Western blot membrane of LC3-II (15 kDa) protein detected with Anti-LC3-II (18725-1-AP, Proteintech) antibody. Gel-separated proteins were transferred to PVDF (0.2 um, millipore). Membranes, incubated with a horseradishperoxidase-conjugated secondary antibody (AS014, ABclonal), were developed with ECL Kit (GK10008, GlpBio). #Weight marker(molecular weight in kDa): SparkJade™ Prestained Protein Ladder, 10 to 180 kDa; catalog number: EC2019.

p62:

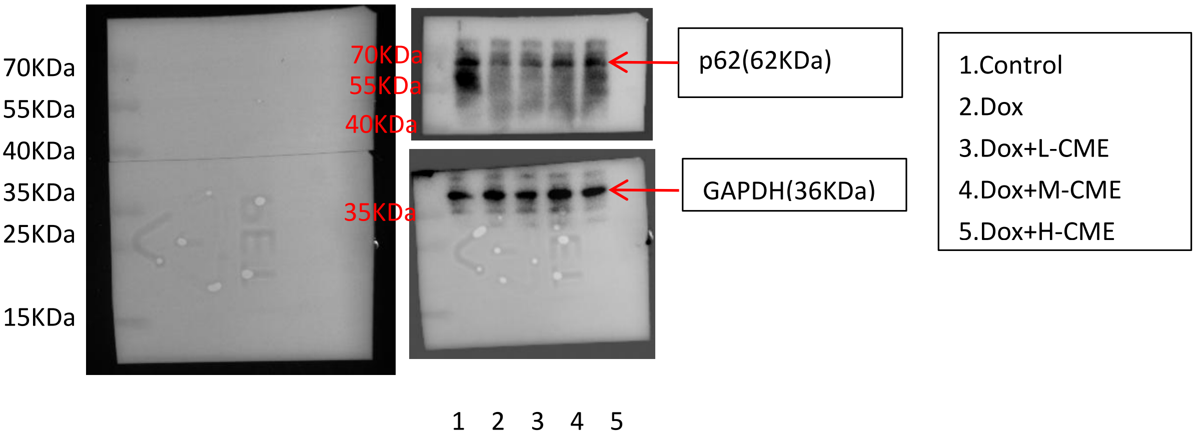

Figure S11. Western blot membrane of p62 (62 kDa) protein detected with Anti-p62 (18420-1-AP, Proteintech) antibody. Gel-separated proteins were transferred to PVDF (0.2 um, millipore). Membranes, incubated with a horseradishperoxidase-conjugated secondary antibody (AS014, ABclonal), were developed with ECL Kit (GK10008, GlpBio). #Weight marker(molecular weight in kDa): SparkJade™ Prestained Protein Ladder, 10 to 180 kDa; catalog number: EC2019.

Figure 6.G Representative WB images in H9c2 cells.

Cleaved caspase3:

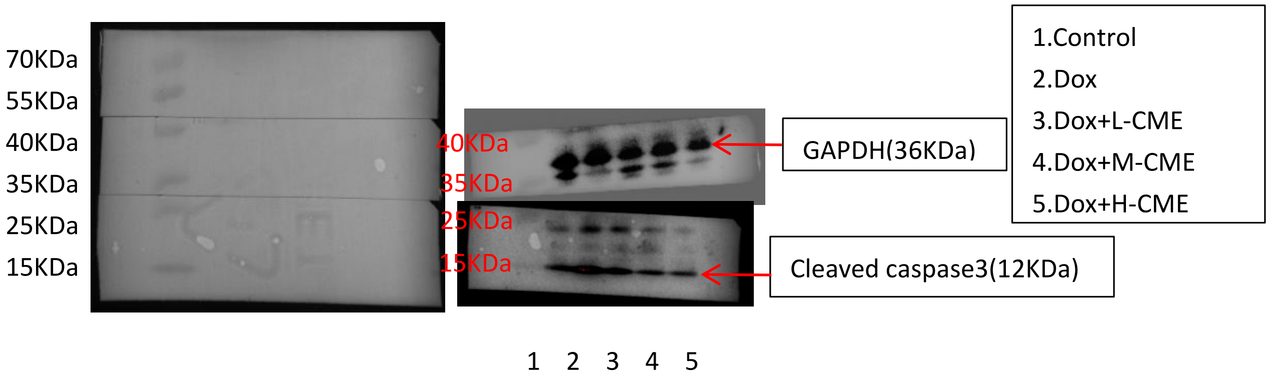

Figure S12. Western blot membrane of Cleaved caspase3 (12 kDa) protein detected with Anti-Cleaved caspase3 (19677-1-AP, Proteintech) antibody. Gel-separated proteins were transferred to PVDF (0.2 um, millipore). Membranes, incubated with a horseradishperoxidase-conjugated secondary antibody (AS014, ABclonal), were developed with ECL Kit (GK10008, GlpBio). #Weight marker(molecular weight in kDa): SparkJade™ Prestained Protein Ladder, 10 to 180 kDa; catalog number: EC2019.

BAX:

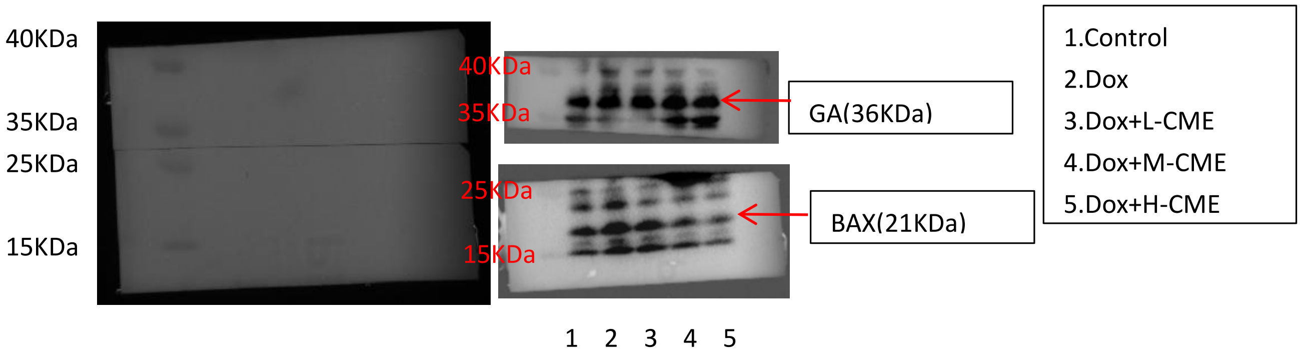

Figure S13. Western blot membrane of BAX (21 kDa) protein detected with Anti-BAX (50599-1-AP, Proteintech) antibody. Gel-separated proteins were transferred to PVDF (0.2 um, millipore). Membranes, incubated with a horseradishperoxidase-conjugated secondary antibody (AS014, ABclonal), were developed with ECL Kit (GK10008, GlpBio). #Weight marker(molecular weight in kDa): SparkJade™ Prestained Protein Ladder, 10 to 180 kDa; catalog number: EC2019.

BCL2:

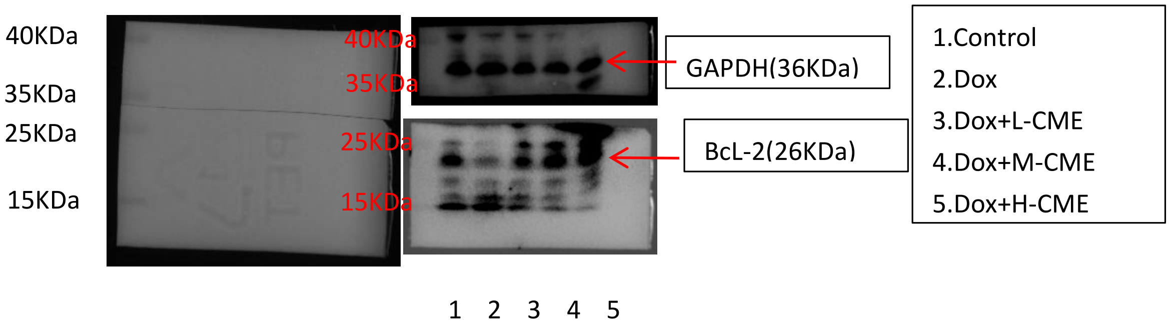

Figure S14. Western blot membrane of Bcl-2 (26 kDa) protein detected with Anti-Bcl-2 (12789-1-AP, Proteintech) antibody. Gel-separated proteins were transferred to PVDF (0.2 um, millipore). Membranes, incubated with a horseradishperoxidase-conjugated secondary antibody (AS014, ABclonal), were developed with ECL Kit (GK10008, GlpBio). #Weight marker(molecular weight in kDa): SparkJade™ Prestained Protein Ladder, 10 to 180 kDa; catalog number: EC2019.

p53:

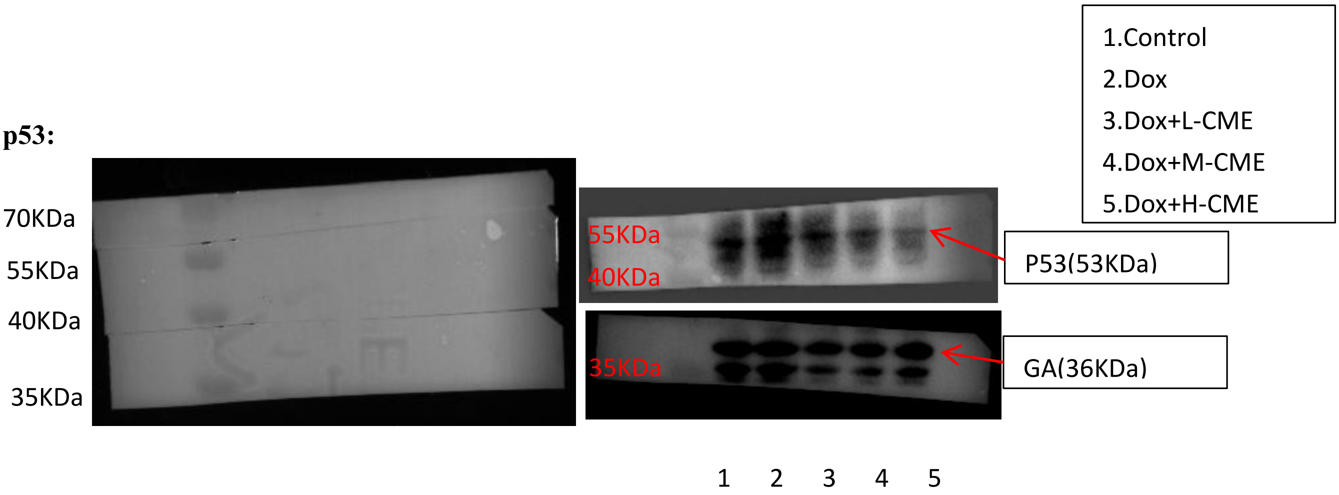

Figure S15. Western blot membrane of p53 (53 kDa) protein detected with Anti-p53 (10442-1-AP, Proteintech) antibody. Gel-separated proteins were transferred to PVDF (0.2 um, millipore). Membranes, incubated with a horseradishperoxidase-conjugated secondary antibody (AS014, ABclonal), were developed with ECL Kit (GK10008, GlpBio). #Weight marker(molecular weight in kDa): SparkJade™ Prestained Protein Ladder, 10 to 180 kDa; catalog number: EC2019.

Beclin-1, LC3-II：

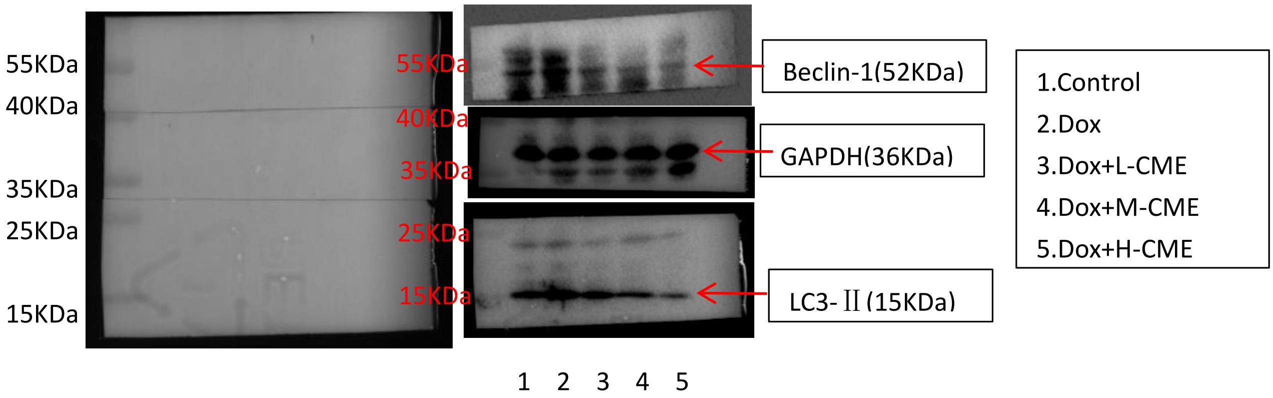

Figure S16. Western blot membrane of Beclin-1 (52 kDa) protein detected with Anti-Beclin-1 (11306-1-AP, Proteintech) antibody. Western blot membrane of LC3-II (15 kDa) protein detected with Anti-LC3-II (18725-1-AP, Proteintech) antibody. Gel-separated proteins were transferred to PVDF (0.2 um, millipore). Membranes, incubated with a horseradishperoxidase-conjugated secondary antibody (AS014, ABclonal), were developed with ECL Kit (GK10008, GlpBio). #Weight marker(molecular weight in kDa): SparkJade™ Prestained Protein Ladder, 10 to 180 kDa; catalog number: EC2019.

p62:

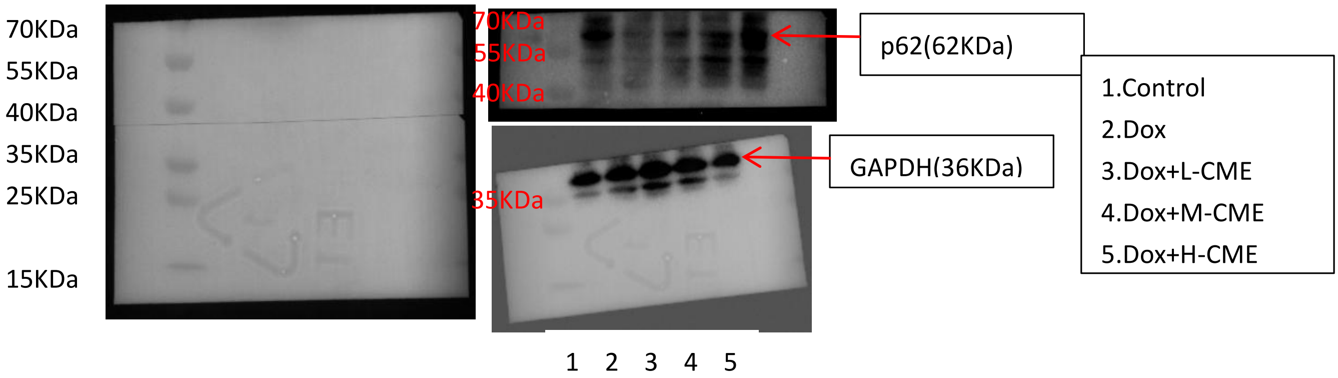

Figure S17. Western blot membrane of p62 (62 kDa) protein detected with Anti-p62 (18420-1-AP, Proteintech) antibody. Gel-separated proteins were transferred to PVDF (0.2 um, millipore). Membranes, incubated with a horseradishperoxidase-conjugated secondary antibody (AS014, ABclonal), were developed with ECL Kit (GK10008, GlpBio). #Weight marker(molecular weight in kDa): SparkJade™ Prestained Protein Ladder, 10 to 180 kDa; catalog number: EC2019.

Figure 6.M Representative WB images in H9c2

cells. **LC3-II：**

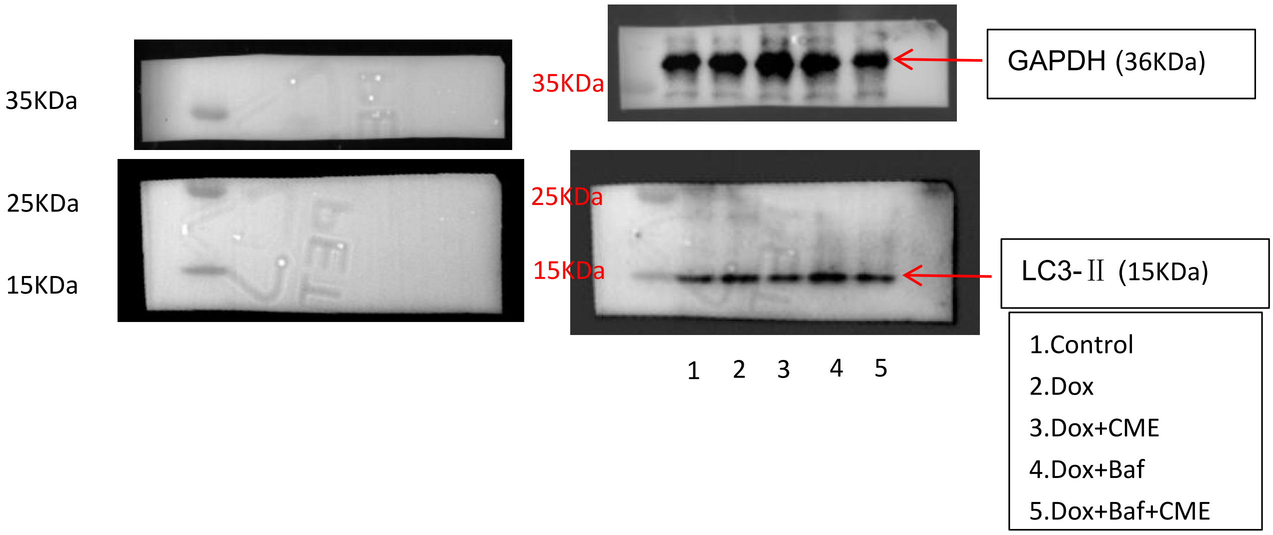

Figure S18. Western blot membrane of LC3-II (15 kDa) protein detected with Anti-LC3-II (18725-1-AP, Proteintech) antibody. Gel-separated proteins were transferred to PVDF (0.2 um, millipore). Membranes, incubated with a horseradishperoxidase-conjugated secondary antibody (AS014, ABclonal), were developed with ECL Kit (GK10008, GlpBio). #Weight marker(molecular weight in kDa): SparkJade™ Prestained Protein Ladder, 10 to 180 kDa; catalog number: EC2019.

Figure 7.C Representative WB images in DIC mice.

TNF-a:

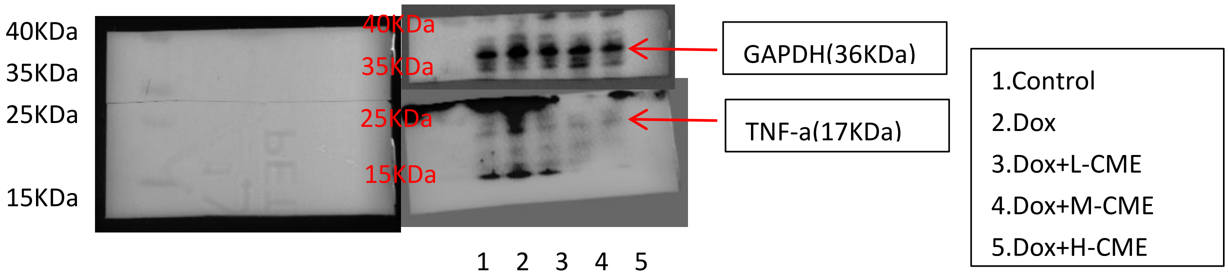

Figure S19. Western blot membrane of TNF-a (17 kDa) protein detected with Anti-TNF-a (346654, Zenbio) antibody. Gel-separated proteins were transferred to PVDF (0.2 um, millipore). Membranes, incubated with a horseradishperoxidase-conjugated secondary antibody (AS014, ABclonal), were developed with ECL Kit (GK10008, GlpBio). #Weight marker(molecular weight in kDa): SparkJade™ Prestained Protein Ladder, 10 to 180 kDa; catalog number: EC2019.

NF-KB and p-NF-KB:

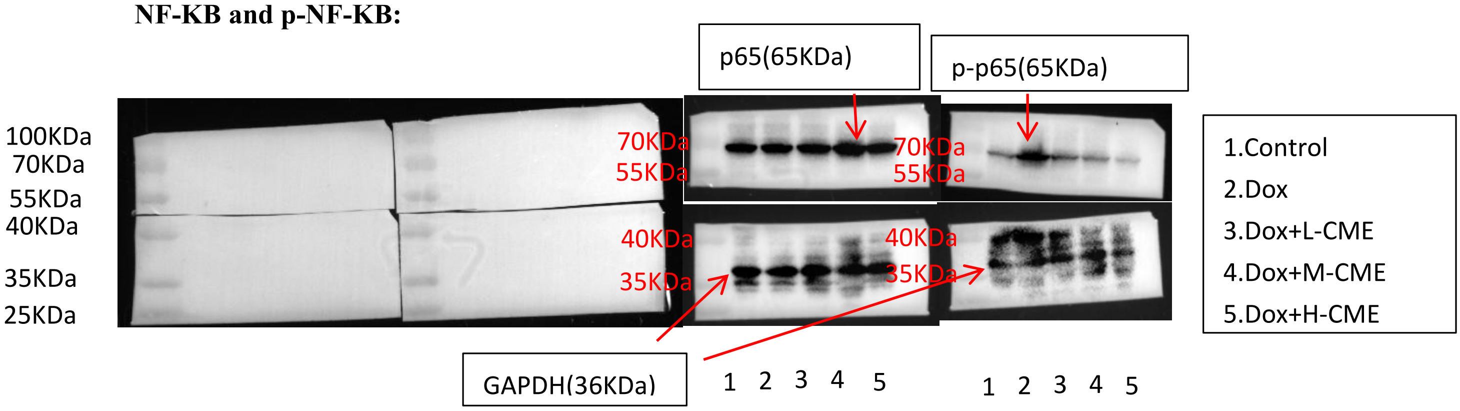

Figure S20. Western blot membrane of NF-κB p65 (65 kDa) protein detected with Anti-NF-κB p65 (F0006, Selleck) antibody. Western blot membrane of Phospho-NF-κB p65 (65 kDa) protein detected with Anti-Phospho-NF-κB p65 (F0155, Selleck) antibody. Gel-separated proteins were transferred to PVDF (0.2 um, millipore). Membranes, incubated with a horseradishperoxidase-conjugated secondary antibody (AS014, ABclonal), were developed with ECL Kit (GK10008, GlpBio). #Weight marker(molecular weight in kDa): SparkJade™ Prestained Protein Ladder, 10 to 180 kDa; catalog number: EC2019.

p38, p-p38:

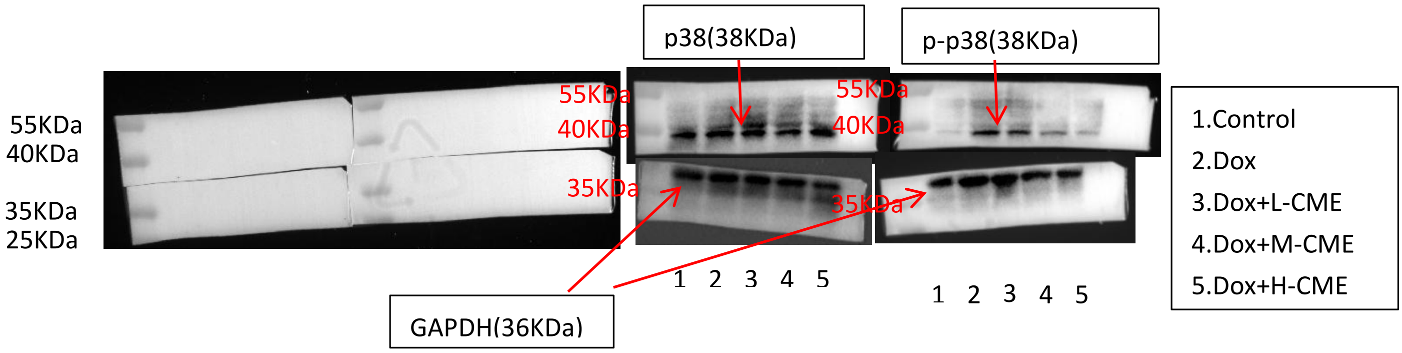

Figure S21. Western blot membrane of p38 (38 kDa) protein detected with Anti-p38 (8690, Cell Signaling Technology) antibody. Western blot membrane of Phospho-p38 (38 kDa) protein detected with Anti-Phospho-p38 (310091, Zenbio) antibody. Gel-separated proteins were transferred to PVDF (0.2 um, millipore). Membranes, incubated with a horseradishperoxidase-conjugated secondary antibody (AS014, ABclonal), were developed with ECL Kit (GK10008, GlpBio). #Weight marker(molecular weight in kDa): SparkJade™ Prestained Protein Ladder, 10 to 180 kDa; catalog number: EC2019.

ERK and p-ERK:

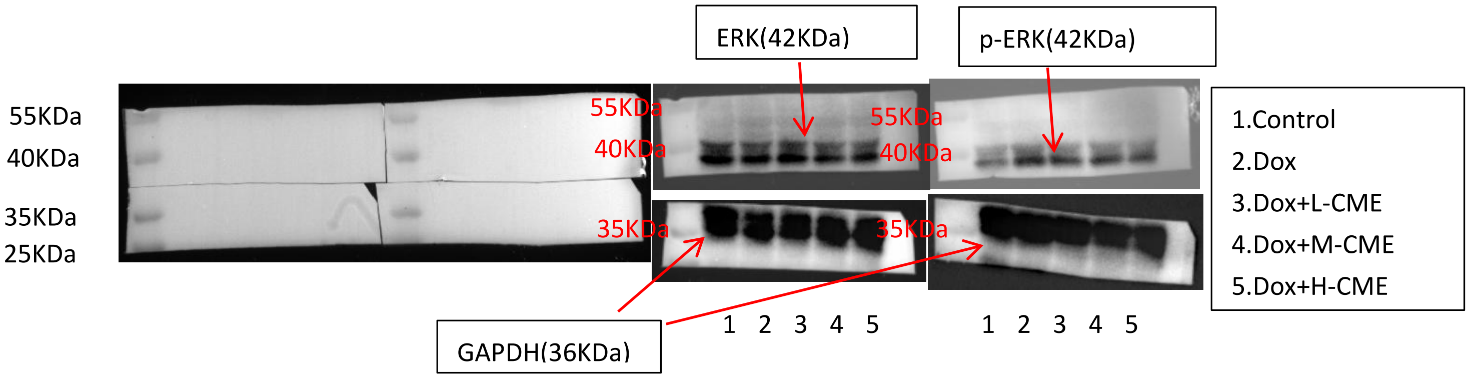

Figure S22. Western blot membrane of ERK (42 kDa) protein detected with Anti-ERK (343830, Zenbio) antibody. Western blot membrane of p-ERK (42 kDa) protein detected with Anti-p-ERK (R24245, Zenbio) antibody. Gel-separated proteins were transferred to PVDF (0.2 um, millipore). Membranes, incubated with a horseradishperoxidase-conjugated secondary antibody (AS014, ABclonal), were developed with ECL Kit (GK10008, GlpBio). #Weight marker(molecular weight in kDa): SparkJade™ Prestained Protein Ladder, 10 to 180 kDa; catalog number: EC2019.

JNK and p-JNK:

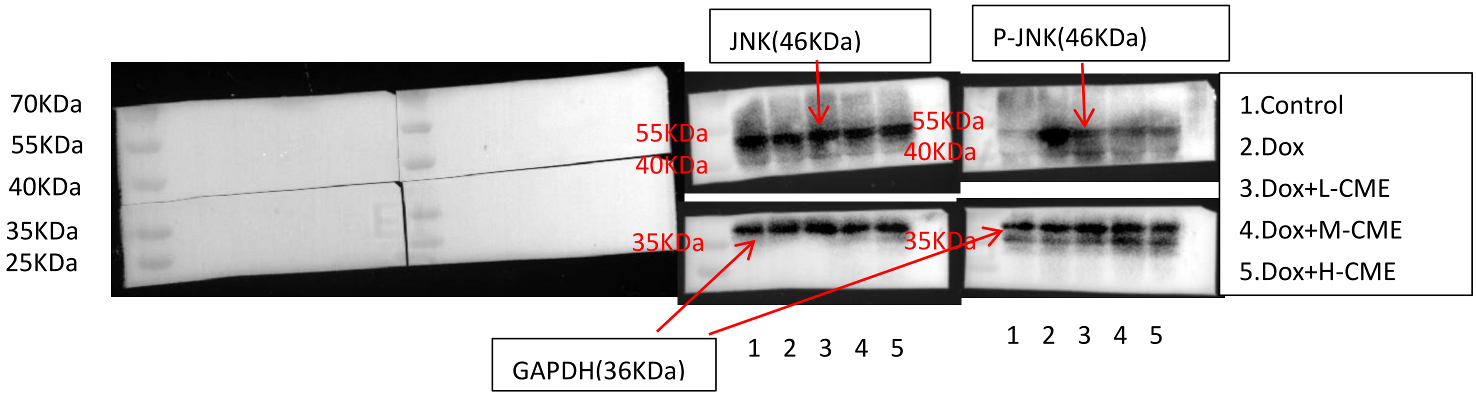

Figure S23. Western blot membrane of JNK (46 kDa) protein detected with Anti-JNK (R22866, Zenbio) antibody. Western blot membrane of p-JNK (46 kDa) protein detected with Anti-p-JNK (R381100, Zenbio) antibody. Gel-separated proteins were transferred to PVDF (0.2 um, millipore). Membranes, incubated with a horseradishperoxidase-conjugated secondary antibody (AS014, ABclonal), were developed with ECL Kit (GK10008, GlpBio). #Weight marker(molecular weight in kDa): SparkJade™ Prestained Protein Ladder, 10 to 180 kDa; catalog number: EC2019.

Figure 7.E Representative WB images in H9c2 cells. TNF-a:

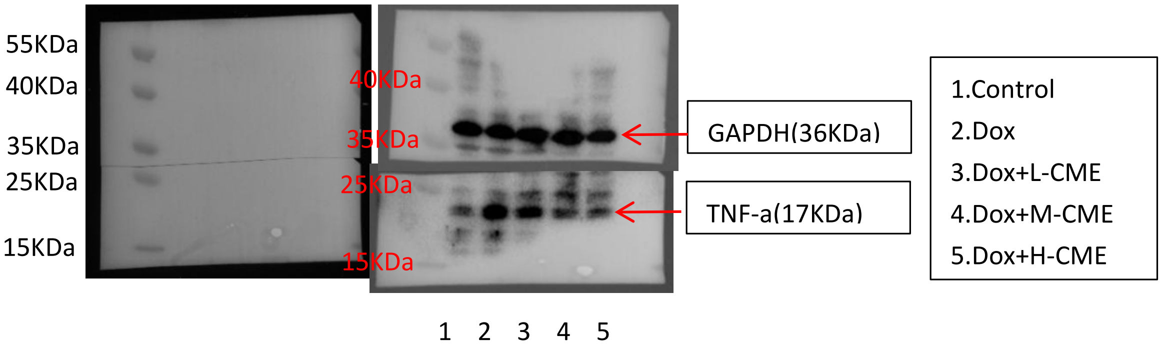

Figure S24. Western blot membrane of TNF-a (17 kDa) protein detected with Anti-TNF-a (346654, Zenbio) antibody. Gel-separated proteins were transferred to PVDF (0.2 um, millipore). Membranes, incubated with a horseradishperoxidase-conjugated secondary antibody (AS014, ABclonal), were developed with ECL Kit (GK10008, GlpBio). #Weight marker(molecular weight in kDa): SparkJade™ Prestained Protein Ladder, 10 to 180 kDa; catalog number: EC2019.

NF-KB p65 and p-NF-KB p65:

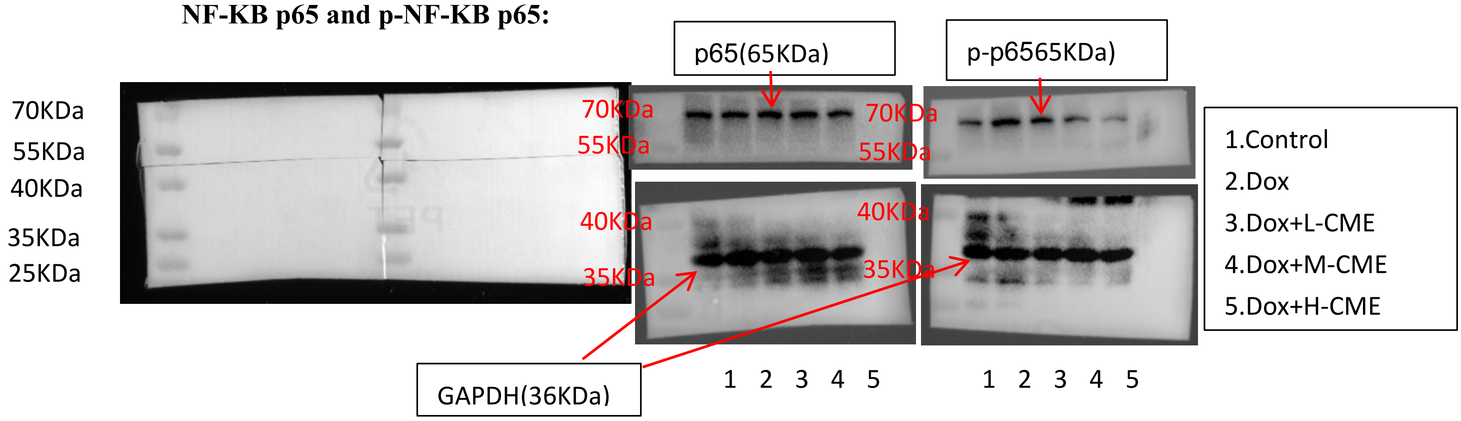

Figure S25. Western blot membrane of NF-κB p65 (65 kDa) protein detected with Anti-NF-κB p65 (F0006, Selleck) antibody. Western blot membrane of Phospho-NF-κB p65 (65 kDa) protein detected with Anti-Phospho-NF-κB p65 (F0155, Selleck) antibody. Gel-separated proteins were transferred to PVDF (0.2 um, millipore). Membranes, incubated with a horseradishperoxidase-conjugated secondary antibody (AS014, ABclonal), were developed with ECL Kit (GK10008, GlpBio). #Weight marker(molecular weight in kDa): SparkJade™ Prestained Protein Ladder, 10 to 180 kDa; catalog number: EC2019.

p38 and p-p38:

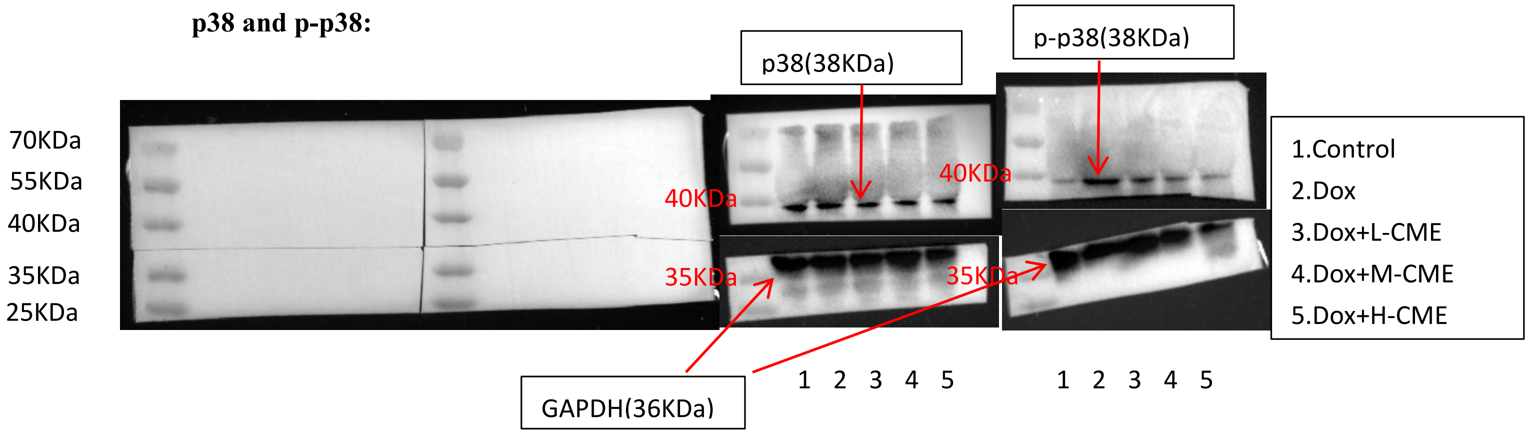

Figure S26. Western blot membrane of p38 (38 kDa) protein detected with Anti-p38 (8690, Cell Signaling Technology) antibody. Western blot membrane of Phospho-p38 (38 kDa) protein detected with Anti-Phospho-p38 (310091, Zenbio) antibody. Gel-separated proteins were transferred to PVDF (0.2 um, millipore). Membranes, incubated with a horseradishperoxidase-conjugated secondary antibody (AS014, ABclonal), were developed with ECL Kit (GK10008, GlpBio). #Weight marker(molecular weight in kDa): SparkJade™ Prestained Protein Ladder, 10 to 180 kDa; catalog number: EC2019.

ERK and p-ERK:

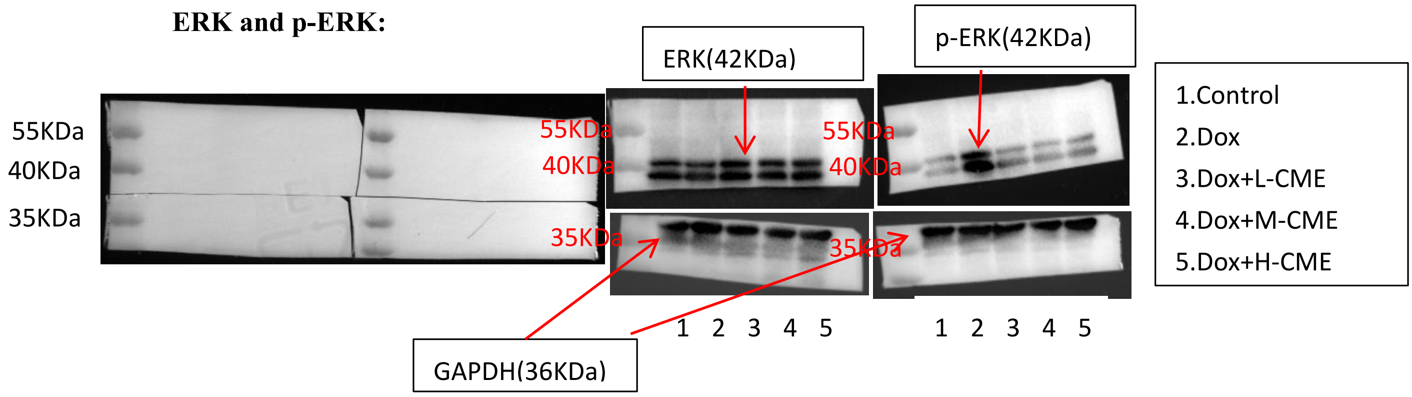

Figure S27 . Western blot membrane of ERK (42 kDa) protein detected with Anti-ERK (343830, Zenbio) antibody. Western blot membrane of p-ERK (42 kDa) protein detected with Anti-P-ERK (R24245, Zenbio) antibody. Gel-separated proteins were transferred to PVDF (0.2 um, millipore). Membranes, incubated with a horseradishperoxidase-conjugated secondary antibody (AS014, ABclonal), were developed with ECL Kit (GK10008, GlpBio). #Weight marker(molecular weight in kDa): SparkJade™ Prestained Protein Ladder, 10 to 180 kDa; catalog number: EC2019.

JNK, p-JNK:

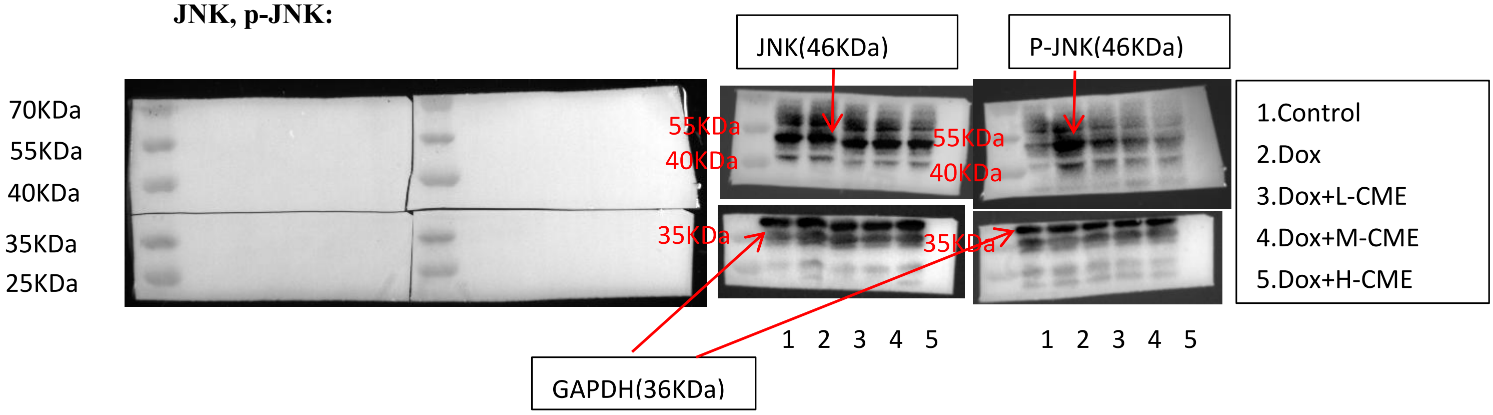

Figure S28. Western blot membrane of JNK (46 kDa) protein detected with Anti-JNK (R22866, Zenbio) antibody. Western blot membrane of p-JNK (46 kDa) protein detected with Anti-p-JNK (R381100, Zenbio) antibody. Gel-separated proteins were transferred to PVDF (0.2 um, millipore). Membranes, incubated with a horseradishperoxidase-conjugated secondary antibody (AS014, ABclonal), were developed with ECL Kit (GK10008, GlpBio). #Weight marker(molecular weight in kDa): SparkJade™ Prestained Protein Ladder, 10 to 180 kDa; catalog number: EC2019.
